# EcDNA-borne structural variants drive oncogenic fusion transcript amplification

**DOI:** 10.1016/j.cell.2025.12.009

**Published:** 2026-01-07

**Authors:** Hyerim Yi, Shu Zhang, Jason Swinderman, Yanbo Wang, Vishnupriya Kanakaveti, King L. Hung, Ivy Tsz-Lo Wong, Suhas Srinivasan, Ellis J. Curtis, Aarohi Bhargava-Shah, Rui Li, Matthew G. Jones, Jens Luebeck, Chris Bailey, Yanding Zhao, Julia A. Belk, Katerina Kraft, Quanming Shi, Xiaowei Yan, Simon K. Pritchard, Kabir S. Mahajan, Frances Liang, Mariam Jamal-Hanjani, Dean W. Felsher, Luke A. Gilbert, Vineet Bafna, Paul S. Mischel, Howard Y. Chang

**Affiliations:** 1 RNA Medicine Program, Stanford University, Stanford, CA, USA; 2 Departments of Dermatology and Genetics, Stanford University School of Medicine, Stanford, CA, USA; 3 Department of Pathology, Stanford University School of Medicine, Stanford, CA, USA; 4 Sarafan ChEM-H, Stanford University, Stanford, CA, USA; 5 Arc Institute, Palo Alto, CA, USA; 6 Department of Urology, University of California, San Francisco, San Francisco, CA, USA; 7 Department of Medicine, Stanford University School of Medicine, Stanford, CA, USA; 8 School of Medicine, University of California at San Diego, La Jolla, CA, USA; 9 School of Medicine, Stanford University, Stanford, CA, USA; 10 Department of Computer Science and Engineering, University of California at San Diego, La Jolla, CA, USA; 11 Cancer Evolution and Genome Instability Laboratory, the Francis Crick Institute, London, UK; 12 Cancer Metastasis Laboratory, University College London Cancer Institute, London, UK; 13 Cancer Research UK Lung Cancer Centre of Excellence, UCL Cancer Institute, London, UK; 14 Department of Medical Oncology, University College London Hospitals, London, UK; 15 Halıcıoğlu Data Science Institute, University of California at San Diego, La Jolla, CA, USA; 16 Present address: Department of Neuroscience, Scripps Research, La Jolla, CA, USA; 17 Present address: Amgen Research, South San Francisco, CA, USA; 18 These authors contributed equally; 19 Lead contact

## Abstract

Extrachromosomal DNA (ecDNA) amplifications are key drivers of human cancers. Here, we show that ecDNAs are major platforms for generating and amplifying oncogene fusion transcripts across diverse cancer types. By integrating analysis of whole-genome and transcriptome sequences from tumor samples and cancer cell lines of a wide variety of tissue types, we reveal that ecDNAs have the highest rate of oncogene fusion events of any copy-number alteration. Focusing on the most common ecDNA fusion hotspot, we find that fusion of the 5′ end of the long noncoding RNA gene, *PVT1—*with exon 1 joined to diverse 3′ partners—confers increased RNA stability, potentially via an SRSF1-dependent mechanism, and enhances MYC-dependent transcription and cancer cell survival. These results demonstrate that ecDNA fosters genome instability and frequent oncogene fusion formation in cancer.

## In brief

Extrachromosomal DNA (ecDNA) is a major source of oncogenic fusions across cancer types, generating tissue-specific fusion landscapes with diagnostic potential. EcDNA-borne *PVT1* 5′-end fusions stabilize partner RNAs and boost oncogene output.

## Graphical Abstract

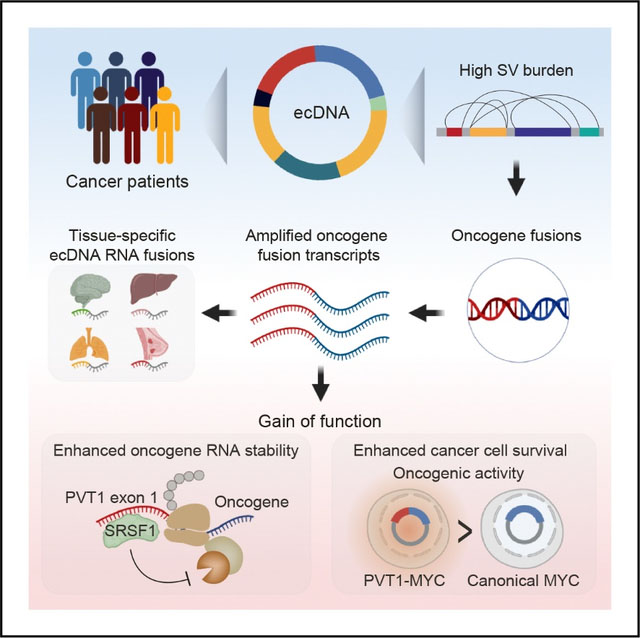

## INTRODUCTION

Extrachromosomal DNAs (ecDNA) are megabase-sized circular episomes encoding oncogenes and additional genetic elements favoring tumor fitness, driving oncogene amplification, intratumoral genetic heterogeneity, rapid tumor evolution, and treatment resistance^1–4^. EcDNA-derived amplifications are detected in 17.1% of tumors across cancer types in adults, with increasing frequency in metastatic tumors, and are associated with shorter survival^3,5,6^. EcDNAs represent a major form of genome instability, driving genomic rearrangements and rapid selection for gain of fitness variants via non-Mendelian inheritance, contributing to poor outcomes for patients.^7–10^ Advanced and metastatic tumors exhibit increased ecDNA copy number and structural complexity,^11,12^ with structural variant (SV) burden particularly elevated within ecDNA-amplified oncogene loci,^13^ underscoring the role of ecDNA-driven genome instability in aggressive cancer progression.

Genomic rearrangements—including translocations, inversions, insertions, and deletions—can generate gene fusions when segments of distinct genes become abnormally joined.^14–17^ Gene fusions are a hallmark of many cancers but are rare in normal tissues.^18^ High SV burden facilitates gene fusions, leading to promoter hijacking or fusion transcript formation, disrupting gene regulation and driving tumorigenesis.^14,19^ Intriguingly, multiple studies demonstrated a strong association between gene fusions and genomic amplifications across cancer types.^20–23^ Recent evidence suggests a potential link between ecDNA and gene fusions, as recurrently truncated genes have been identified on ecDNA.^24^ However, the role of ecDNA in driving SVs that generate gene fusions and their functional consequences remains unclear.

Here, we systematically investigated the landscape of gene fusions associated with ecDNA, demonstrating that ecDNA-driven genome instability is a major source of oncogene fusions in human cancer. We further focus on the most common ecDNA fusion hotspot, *Plasmacytoma Variant Translocation 1* (*PVT1*), revealing potential mechanisms by which ecDNA-dependent fusions can promote oncogenic regulations in cancer.

## RESULTS

### EcDNA is a major platform for generating and amplifying oncogene RNA fusions in cancer

**Figure 1.**
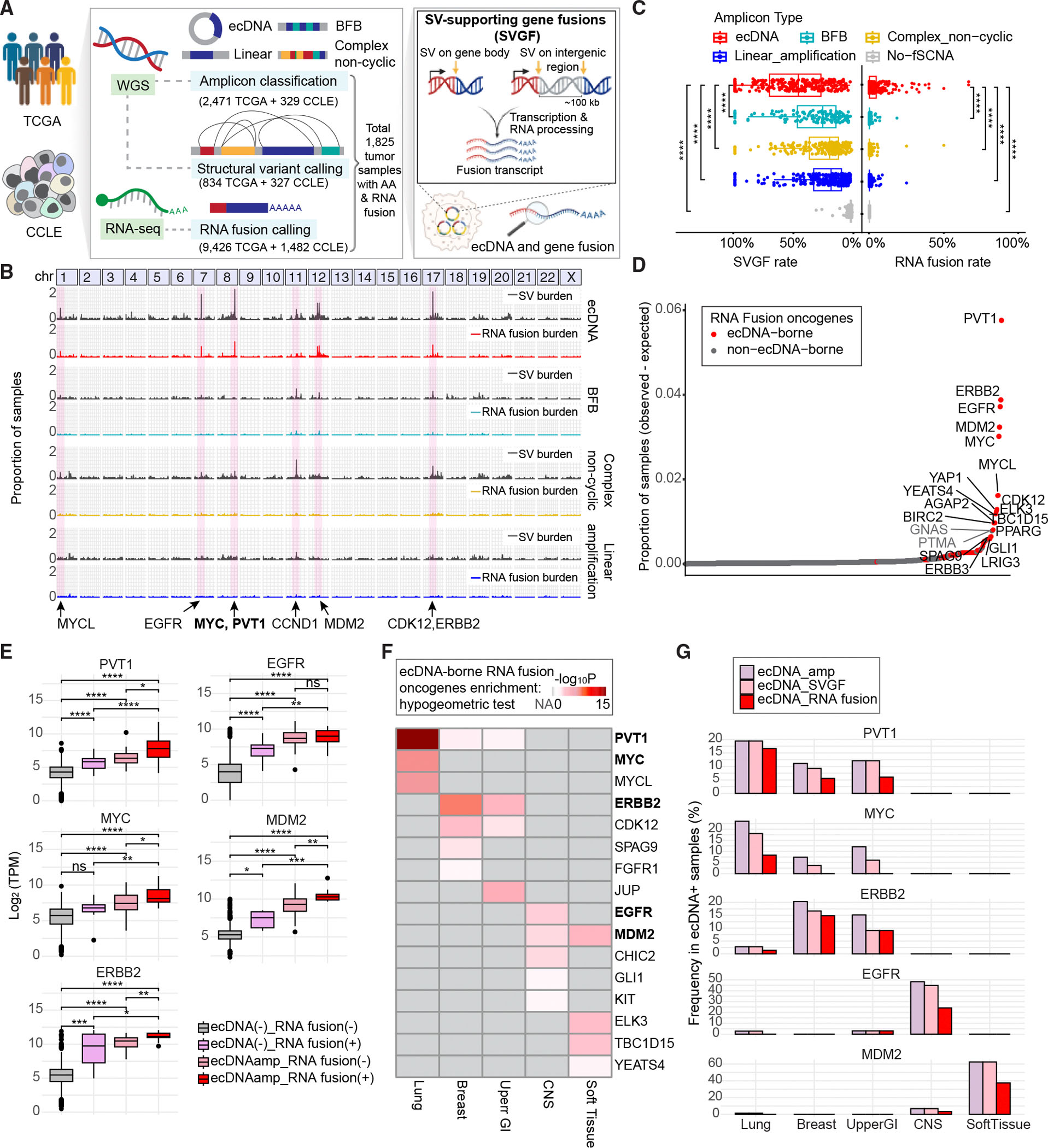
Landscape of oncogene fusions on ecDNAs (A) Schematic of the analysis workflow for TCGA and CCLE databases. ecDNA, extrachromosomal DNA; BFB, breakage-fusion bridge; No-fSCNA, no focal somatic copy-number amplification. (B) Genome-wide distribution of SV and RNA fusion burden by amplicon type. (C) SVGF and RNA fusion rate across amplicon types. Each dot represents an amplicon interval in a cancer sample. (D) Enrichment of oncogene RNA fusions, ranked by the difference between the observed and expected proportion of cancer samples with either ecDNA-borne RNA fusions (red) or non-ecDNA-borne RNA fusions (gray). Each dot represents an RNA fusion oncogene. (E) Transcript levels of representative ecDNA-borne RNA fusion oncogenes grouped by RNA fusions and ecDNA amplification status. (F) Enrichment scores of ecDNA-borne RNA fusion oncogenes across cancer types. Color of scale bar: gray “not applicable” (NA; <5 ecDNA[+] samples), white “not significant,” and pink to red “significance of enrichment.” (G) Percentage of ecDNA(+) cancer samples with amplification, SVGF, or RNA fusions of representative oncogenes in each cancer type. (C and E) *p* > 0.05 (ns), *p < 0.05, **p < 0.01, ***p < 0.001, ****p < 0.0001 by two-tailed unpaired *t* test. See also Figure S1 and Tables S1 and S2.

To investigate the association between gene fusions and different amplicon types, we integrated Amplicon Architect (AA) data from whole-genome sequencing (WGS) and RNA fusion data from RNA sequencing (RNA-seq)^25^ for 1,825 matched samples across 83 cancer types from The Cancer Genome Atlas (TCGA) and Cancer Cell Line Encyclopedia (CCLE) databases, enabling systematic and comprehensive annotation of amplicon types to the breakpoints of 525,648 SVs and 23,546 RNA fusions (Figure 1A; Table S1). As fusion transcripts serve as direct evidence of gene fusions, we analyzed the co-occurrence of SV (SV burden^13^) and RNA fusion breakpoints (RNA fusion burden) in 100 kb genomic windows across the genome and found strong alignment between RNA fusion and SV burden peaks (Figure 1B). SV and RNA fusion burden were more strongly correlated on ecDNA (*R* = 0.7) than other focal amplicon types (*R* ≤ 0.56) or no focal somatic copy-number amplification (No-fSCNA^5^) (R = 0.08) (Figure S1A). Notably, 88.5% of RNA fusions from ecDNA-amplified genes—hereafter referred to as ecDNA-borne RNA fusions—were supported by SVs. Across all cancers analyzed (*n* = 1,825), samples harboring ecDNA-borne RNA fusions accounted for 14.5% (264/1,825). Among ecDNA-positive cancers (26.2%, 479/1,825), more than half (55.1%, 264/479) harbored ecDNA-borne RNA fusions, underscoring the strong link between ecDNAs and fusion transcript generation.

Fusion transcripts can arise when SVs occur within gene bodies or in intergenic regions, repositioning two genes adjacent to each other and leading to fusion after transcription and RNA processing.^17^ We defined SVs that contribute to fusion transcript formation as SVGF (SVs supporting gene fusions) based on their breakpoint locations—either within gene bodies or in intergenic regions near a gene body (~100 kb)^26–28^ (Figure 1A). The SVGF rate, defined as the proportion of genes with SVGF relative to all genes within each amplicon interval, was highest in ecDNA compared with other focal amplifications and No-fSCNA (Figure 1C), indicating that ecDNA is more prone to SV-mediated gene fusions. Consistently, ecDNA exhibited the highest RNA fusion frequency within each amplicon interval, among any other copy-number alteration types (Figure 1C). Using long-read DNA and RNA-seq in ecDNA-positive cell line models, we validated precise SV breakpoints on ecDNA encoding highly expressed fusion transcripts across multiple cell lines (Figure S1B; Table S1). Notably, the top expressed fusion transcripts in each cell line almost always originated from ecDNA-borne gene fusions. Consistently, ecDNA-borne fusion transcripts showed higher expression than non-ecDNA-derived fusions in cancers (Figure S1C), reinforcing ecDNA as a hotspot for SV-driven gene fusions and amplification of fusion transcript expression.

EcDNAs are more prone to harbor activating mutations that confer selective advantages to cancer cells, facilitating positive selection.^9,10,29,30^ To identify specific gene fusions associated with ecDNA, we mapped SV and RNA fusion burden peaks across the genome (Figure 1B). In ecDNA-positive cancers, both SV and RNA fusion burdens peaked at well-known ecDNA-amplified oncogenes such as *EGFR*, *MYC/PVT1*, *MDM2*, and *ERBB2* (Figure 1B). The most frequent ecDNA-borne fusions originated from these amplified oncogenes,^6,24^ whereas non-ecDNA-borne RNA fusions were enriched in distinct oncogenes not commonly amplified on ecDNA (Figure S1D). Comparing observed versus expected probabilities (STAR Methods), ecDNA-borne RNA fusion samples showed strong preferential enrichment for oncogene RNA fusions (Figure 1D).

To investigate the functional relationship between oncogene RNA fusions and ecDNA, we examined RNA expression of the top 5 enriched ecDNA-borne fusion oncogenes (Figure 1E) by comparing groups categorized by RNA fusions and ecDNA amplification status. Most oncogenes showed higher expression in RNA fusion-positive versus RNA fusion-negative samples, with the highest expression observed in those harboring both RNA fusions and ecDNA amplification, which was strongly supported by DNA copy-number gains (Figures 1E and S1E). Within RNA fusion-positive samples, higher expression in ecDNA-positive samples was largely driven by copy-number amplification, underscoring ecDNA’s role in upregulating oncogene fusion expression.

Given that oncogene amplification on ecDNA is closely linked to tissue contexts,^6^ we next profiled ecDNA-borne oncogene RNA fusions across cancer types. These fusion transcripts showed distinct tissue enrichment (Figure 1F), with *PVT1* and *MYC* most enriched in lung cancer, *ERBB2* in breast and upper GI cancers, *EGFR* in CNS tumors, and *MDM2* in soft tissue sarcomas (Figure 1F; Table S2). Consistent with the strong association between ecDNA amplification, SVs, and fusion RNA expression (Figures 1B and S1A), these oncogene RNA fusions were supported by both ecDNA amplification and SVGFs in a cancer type-specific manner (Figure 1G). Collectively, our data demonstrate that structural rearrangements on ecDNA produce the highest rate of oncogene fusions of any copy-number alterations, driving their elevated expression across cancers.

### Landscape of ecDNA-borne *PVT1* fusions

To investigate how ecDNA-derived fusions contribute to tumor pathogenesis, we focused on the long non-coding RNA, *PVT1*, the most frequent fusion hotspot in ecDNA-positive cancers. *PVT1* fusion was 4.4-fold more enriched in ecDNA-borne than non-ecDNA-borne RNA fusion cancers (Figure S1D). In ecDNA-positive cancers, the *MYC/PVT1* locus exhibited the highest SV and RNA fusion burden across the cancer genome (Figure 1B). Originally identified as a breakpoint hotspot in Burkitt’s lymphomas,^31^ *PVT1* suffers frequent gene fusion involving multiple partners across cancers.^14,32,33^ Consistent with its genomic fragility and highest SV and RNA fusion burden in ecDNA-positive samples, *PVT1* produced the greatest diversity of fusion transcript, with 72.3% arising from ecDNA (Figure S1F). Moreover, when amplified on ecDNA, *PVT1* generates even more fusion variants (Figure S1G). Overall, oncogenes on ecDNA tended to produce more diverse fusion species than those on other amplicon types, highlighting ecDNA’s role in promoting oncogene RNA fusions.

**Figure 2.**
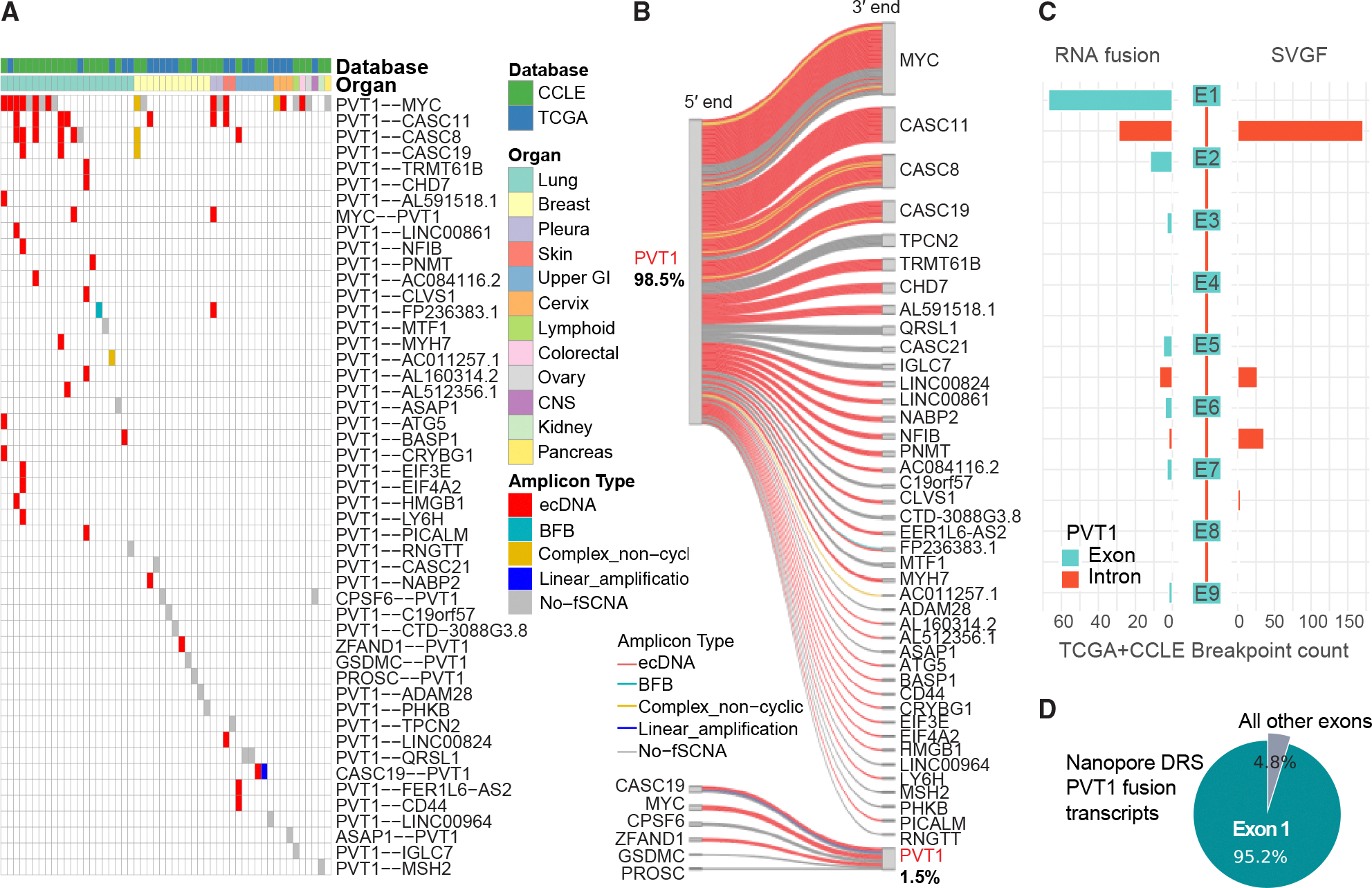
*PVT1* 5′ end is the most prevalent RNA fusion enriched on ecDNA (A) Distribution of *PVT1* fusion transcripts across cancer types, annotated by amplicon type. Rows correspond to *PVT1* fusion transcript species, and columns to cancer samples, clustered by database and cancer type (organ) with amplicon types as color-coded bars. (B) Position of *PVT1* (5′ or 3′ end) in *PVT1* fusion transcripts. Each line represents a distinct fusion transcript with unique RNA breakpoints, color-coded by amplicon types. (C) Distribution of RNA fusion breakpoints (left) and SVGF breakpoints (right) for *PVT1* fusions across exonic and intronic regions of the *PVT1* gene. (D) Proportion of *PVT1* fusion transcripts containing *PVT1* exon 1, measured by long-read RNA-seq in ecDNA(+) cell line models and their isogenic pairs where *PVT1* fusion transcripts are detected. (A–C) Samples are from TCGA and CCLE. See also Figure S2 and Tables S1 and S2.

Given that *PVT1* forms fusions with multiple partners^33^ and its ecDNA-borne RNA fusions exhibit tissue specificity (Figures 1F and 1G), we profiled RNA expression of *PVT1* fusions across cancer types and association with amplicon types. *PVT1* RNA fusions were detected broadly across cancers, involving multiple oncogene partners, with the most prevalent forms having oncogenes adjacent to the *PVT1* locus (Figures 2A and S2A). Diverse ecDNA-borne *PVT1*-fusion transcripts were observed predominantly in lung cancers (65%, 13/20), suggesting extensive genomic rearrangements surrounding the *PVT1* locus on ecDNA in this cancer type (Figure 2A; Table S2). AA analysis confirmed dynamic rearrangements on the *PVT1* locus generating multiple fusions (*MYC*, *CASC8*, and *MYH7*) in ecDNA-positive lung cancer cell lines (Figure S2B). Notably, ecDNA-borne *PVT1* fusions occurred in 10.6% of small cell lung cancer (SCLC) cell lines, whereas lung adenocarcinoma (LUAD) showed 2.9% (Figure S2C), indicating cancer-subtype specificity. By contrast, in breast cancers, *PVT1* fusions were less frequently ecDNA-associated (0.8%) and more linked to No-fSCNA, suggesting tissue-dependent amplicon preference in *PVT1* fusion formation (Figures 2A and S2C).

*PVT1* gene*—*located 55 kb downstream of the *MYC* oncogene on human chromosome 8q24^34,35^—encodes a long noncoding RNA (lncRNA), expressed at low levels in normal tissues, but upregulated in many cancers, where it is associated with tumor onset, progression, and poor prognosis.^32,33,36,37^ Multiple studies have linked *PVT1* fusions to 8q24 copy-number amplification,^4,33,38,39^ involving co-amplification of *MYC* and *PVT1*. For instance, *PVT1-MYC* fusions are detected in 60% of *MYC*-amplified group 3 medulloblastomas, characterized by *MYC* ecDNA amplification.^39–41^ Notably, all *MYC* fusion transcripts in Figures 1E and S1E were fused to *PVT1*. The prevalent *PVT1*-fusion partners—*MYC*, *CASC11*, *CASC8*, and *CASC19* at 8q24—are frequently co-amplified on ecDNA (Figures 2A, 2B, and S2A), with *PVT1-MYC* as the most prevalent ecDNA fusion (Table S1). *PVT1* fusion transcript levels were significantly higher in cancers with ecDNA-borne RNA fusions compared with those with non-ecDNA-borne RNA fusions (Figure S2D). This pattern was consistently observed for *PVT1*-fused *MYC*, *CASC11*, and *CASC8*, all supported by ecDNA-driven copy-number gain (Figures 1E, S1E, and S2E). This strong association between *PVT1* fusion and ecDNA underscores *PVT1* fusion transcripts as biomarkers for ecDNA-positive cancers. Indeed, combining *PVT1* fusion expression with *PVT1* copy-number gain (CN > 10) improved ecDNA detection accuracy to 95%, compared with 74.5% or 82.1% using either metric alone (Figure S2F).

Integrated analysis of TCGA and CCLE showed that 98.5% of *PVT1* fusion transcripts have *PVT1* at their 5′ end (Figure 2B), consistent with prior reports.^33^ Recurrent RNA fusion and SV breakpoints are concentrated in *PVT1* exon 1 and intron 1, respectively, generating mature transcripts that retain exon 1 of lncRNA *PVT1* (Figure 2C). SV breakpoint analysis revealed that *PVT1* fusions rarely disrupt the promoter or exon 1, with no breakpoints of ecDNA-borne *PVT1* fusions detected within these regions (Figure S2G). Consistent with this, these elements are always co-amplified in cancers with ecDNA-borne *PVT1* fusions (Figure S2H). Precise mapping by long-read sequencing confirmed that *PVT1* fusion breakpoints occur most frequently in exon 1 and intron 1, with 95.2% of *PVT1* fusion transcripts harboring exon 1 (Figures 2C, 2D, and S2I). Although exon 1 comprises only 7%–12% of *PVT1* lncRNA isoforms, it is retained in 95% of the amplified fusion transcript, an 8–14-fold enrichment indicative of positive selection. Such recurring mutational “hotspot” suggests gain-of-function activity^42^ for *PVT1* exon 1 in tumor evolution, likely facilitated by the asymmetric inheritance of ecDNA. Together, these results suggest ecDNA-mediated genomic rearrangements as a key driver of 5′ *PVT1* exon 1 fusions, a predominant oncogene fusion form in cancer.

### *PVT1* exon 1 fusion enhances mRNA stability

**Figure 3.**
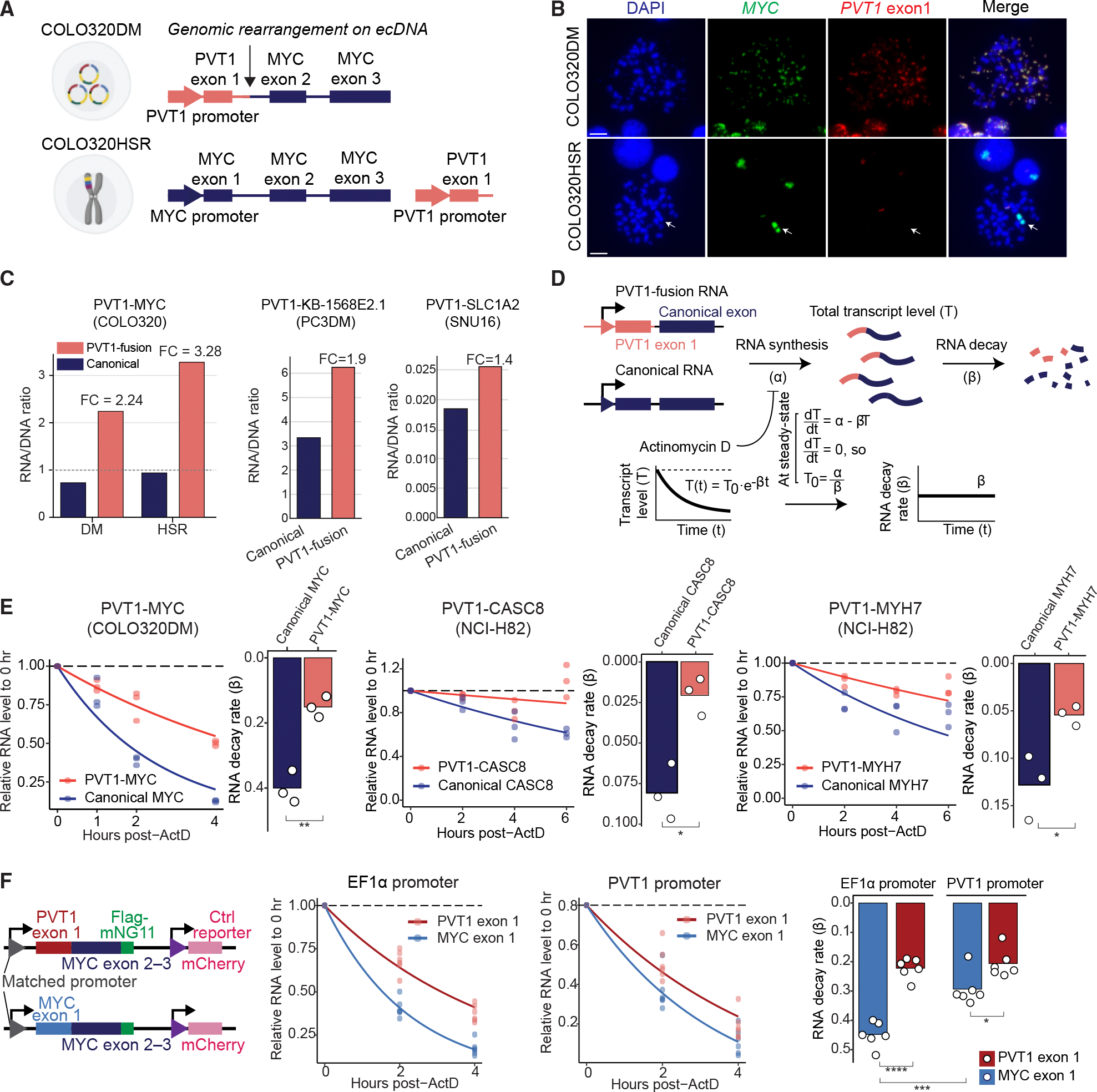
*PVT1* exon 1 fusion enhances RNA stability (A) Schematic of ecDNA rearrangement in COLO320DM generating the *PVT1-MYC* fusion and canonical *MYC* in COLO320HSR. (B) Metaphase DNA FISH images of *MYC* and *PVT1* exon 1 in COLO320DM and COLO320HSR. Scale bars, 10 μm. (C) RNA/DNA ratio of *PVT1* fusion transcripts in ecDNA(+) cell lines, measured by long-read sequencing. Fold change (FC) relative to canonical transcripts is indicated. (D) Schematic of RNA stability assay and modeling for RNA decay rate (*β*). (E) RNA stability and decay rates of endogenous *PVT1-*fusion and canonical transcripts after actinomycin D (ActD) treatment (*n* = 3). (F) RNA stability and decay rates of reporter transcripts in COLO320DM after ActD treatment (*n* = 6). Left: schematic of reporters. Middle: RNA stability measurement. Right: RNA decay rates. (E and F) *p < 0.03, **p < 0.002, ****p < 0.0001 by two-tailed *t* test. RNA abundance was measured by RT-qPCR and normalized to 0 h and *GAPDH* internal control RNA. See also Figure S3 and Table S3.

To investigate the molecular function of *PVT1* exon 1 fusion, we utilized a cell line model, COLO320DM, carrying *PVT1-MYC* on ecDNA, the most prevalent form of *PVT1* fusion (38.5%; 20/52). In COLO320DM, the *MYC* promoter and exon 1 are replaced by the *PVT1* promoter and exon 1 on ecDNA (Figure 3A), producing a mature *PVT1-MYC* fusion mRNA, where *PVT1* exon 1 joins *MYC* exons 2–3. Intriguingly, in the near-isogenic pair COLO320HSR line, generated by chromosomal integration of ecDNA,^43^ *MYC* is amplified on the chromosome, not the ecDNA, and neither amplification nor fusion of the *PVT1* 5′ region is observed,^4^ as confirmed by metaphase DNA fluorescence *in situ* hybridization (FISH). In COLO320DM, *PVT1* exon 1 and *MYC* FISH signals colocalize predominantly on ecDNA, whereas colocalization was rare on homogenous staining region (HSR) chromosomes in COLO320HSR (Figure 3B). Occasional colocalization at rare linear integration sites was observed but absent in canonical HSR regions, resulting in *PVT1-MYC* fusion being largely absent in COLO320HSR (Figure S3A). This ecDNA-specific fusion, together with the shared genetic background of COLO320DM and COLO320HSR, makes this isogenic pair an ideal model to dissect *PVT1* fusion function.

To investigate the impact of *PVT1* fusion on gene expression, we compared DNA copy-number and RNA expression levels of *PVT1*-fusion and canonical isoforms using long-read DNA and RNA-seq, respectively, precisely identifying and quantifying each isoform. Consistent with WGS and DNA FISH imaging data, *PVT1-MYC* dominated in COLO320DM, comprising 60% of total *MYC* DNA copies and 82% of total *MYC* transcripts, while canonical *MYC* was the predominant isoform in COLO320HSR, occupying 96% of DNA copies and 87% of transcripts (Figure S3B). These findings support the preferential association of *PVT1-MYC* fusion with ecDNA (Figures 2A and 2B). In TCGA and CCLE cancer databases, *PVT1-MYC* RNA fusion was 6-fold enriched with ecDNA-borne RNA fusions (3.9%; 10/264) compared with those with non-ecDNA-borne RNA fusion (0.6%; 10/1,561). Notably, after normalizing for DNA copy number, steady-state *PVT1-MYC* mRNA level was ~2–3-fold higher than that of canonical *MYC* mRNA in both COLO320DM and COLO320HSR cells (Figure 3C), indicating that *PVT1*-fusion elevates RNA expression beyond gene dosage. Similar elevation was observed for other *PVT1*-fusion transcripts across multiple cancer types (Figure 3C), suggesting a general mechanism of RNA upregulation by *PVT1* fusions.

To understand the underlying mechanism of how *PVT1* exon 1 fusion increases RNA expression of its fusion partner, we developed luciferase reporter constructs (nLuc) with or without *PVT1* exon 1 under the matched promoter to control for transcription efficiency (Figure S3C). Steady-state reporter RNA levels were assessed by normalizing to an internal control reporter RNA (fLuc), which was expressed under a separate promoter on the same plasmid, to account for transfection efficiency. *PVT1* exon 1 fusion resulted in a 2–3-fold increase in reporter steady-state mRNA levels compared with the non-fused reporter under the same promoter (minimal promoter^4^) (Figure S3C), suggesting that post-transcriptional mechanisms contribute to the elevated RNA level of *PVT1-*fusion transcripts. Furthermore, combining *PVT1* exon 1 fusion with the *PVT1* promoter led to an even greater increase in reporter RNA levels (Figure S3C). These results suggest that the *PVT1* 5′ end, including the promoter and exon 1, which represents the predominant *PVT1* fusion pattern across cancers (Figures S2G and S2H), functions as a distinct regulatory unit.

Notably, COLO320DM, but not COLO320HSR, was shown to express an aberrant *MYC* transcript isoform lacking *MYC* exon 1, with greater RNA stability.^44^ Our RNA long-read sequencing data confirmed that *PVT1-MYC*, which replaces *MYC* exon 1 with *PVT1* exon 1, is the predominant *MYC* transcript isoform in COLO320DM, whereas canonical *MYC* dominates in COLO320HSR (Figure S3B). To test whether *PVT1* exon 1 fusion regulates RNA stability, we measured the RNA half-life of endogenous *PVT1* fusion and their canonical RNAs across multiple partner genes and cell lines, including lung cancers where *PVT1* fusion is most prevalent. RNA decay rates were estimated based on the observed RNA abundance changes upon transcription inhibition with actinomycin D using mathematical modeling (Figure 3D; STAR Methods). *PVT1*-fusion transcripts consistently exhibited higher RNA stability compared with their canonical counterparts across multiple cell lines (Figures 3E, S3D, and S3E), suggesting a general role of *PVT1* exon 1 in RNA stabilization. Notably, the 2–3-fold slower RNA decay rates of *PVT1-MYC* compared with canonical *MYC* closely matched the fold difference in steady-state endogenous RNA levels (Figure 3C), supporting the role of *PVT1* exon 1-mediated RNA stabilization in RNA upregulation.

To test whether *PVT1* exon 1 confers RNA stability, we generated reporter constructs in which either *PVT1* exon 1 or *MYC* exon 1 was fused to *MYC* exon 2–3-FLAG-mNG11, under the matched promoter (*EF1α* or *PVT1*) to mimic endogenous conditions (Figure 3F). Similar to luciferase-based reporter assays (Figure S3C), the *PVT1* exon 1-fused *MYC* reporter exhibited higher steady-state RNA expression than the *MYC* exon 1-fused reporter, with a further increase when combined with the *PVT1* promoter (Figure S3F). Under the control of transcription efficiency with the matched promoters, *PVT1* exon 1-fused reporter showed higher RNA stability (Figure 3F), demonstrating that *PVT1* exon 1 confers RNA stability.

### SRSF1 binding contributes to *PVT1* exon 1-mediated RNA stabilization

**Figure 4.**
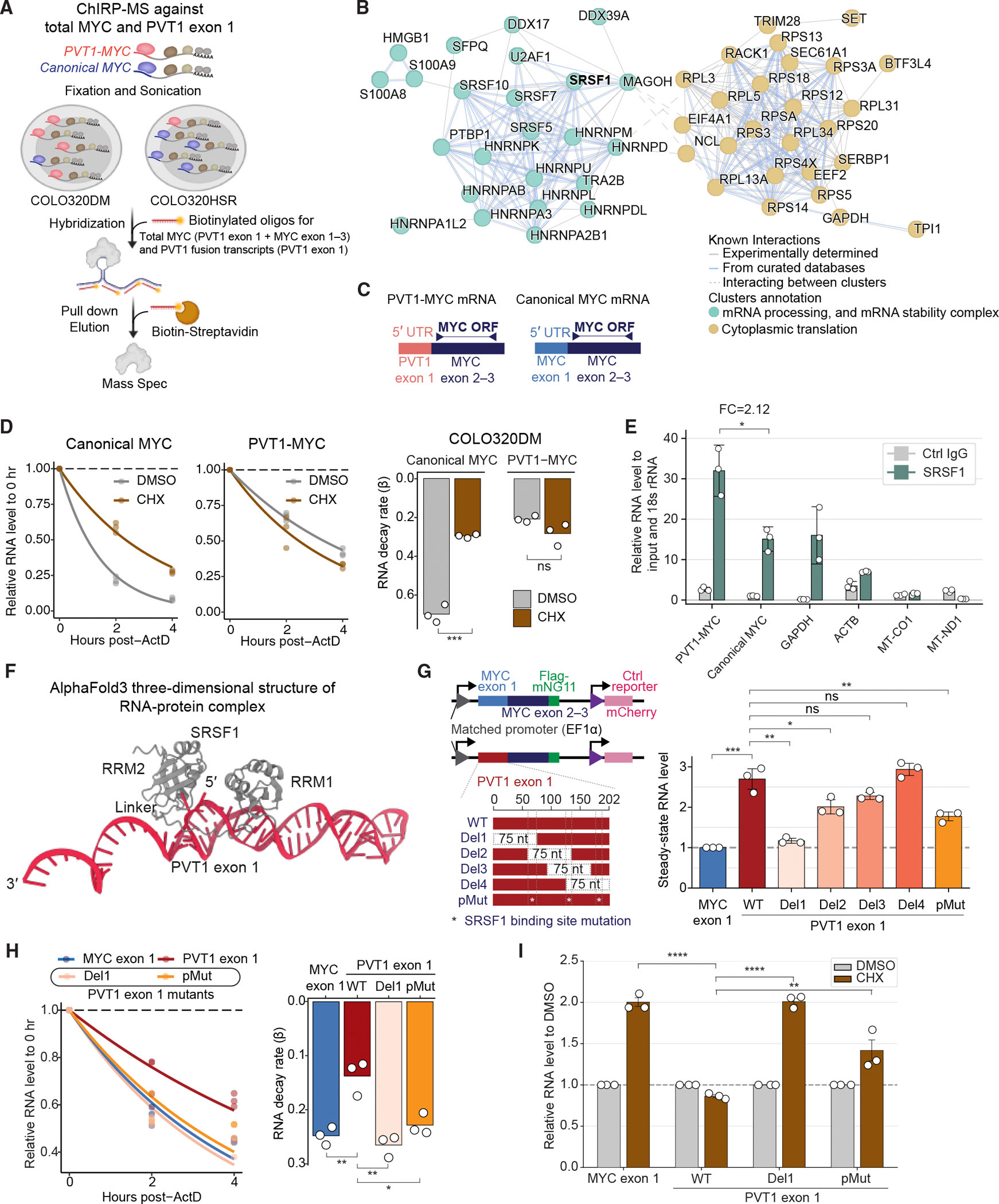
SRSF1 binding contributes to *PVT1* exon 1-mediated RNA stabilization (A) Schematic of ChIRP-MS. (B) Functional clustering of *PVT1*-*MYC* ChIRP-MS hits. Nodes, RNA-binding proteins (RBPs); node colors, clusters; solid lines, RBP interactions; dotted lines, interactions between clusters. (C) Schematic of *PVT1-MYC* and canonical *MYC* mRNAs. (D) RNA stability and decay rates of endogenous *PVT1-MYC* and canonical *MYC* mRNAs after cycloheximide (CHX) and ActD in COLO320DM (*n* = 4). Line: RNA stability measurement. Bar: RNA decay rates. (E) Endogenous RNA enrichment of SRSF1 RNA-IP in COLO320DM cells (*n* = 3). Enrichment is calculated relative to input RNA levels and 18S rRNA. Mitochondrial mRNAs (*MT-CO1* and *MT-ND1*) served as negative controls. (F) AlphaFold3-predicted structure of the interaction between SRSF1 RNA recognition motifs (RRMs) and *PVT1* lncRNA exon 1 (nucleotides 62–141). (G) Steady-state reporter RNA levels in HEK293T cells (*n* = 3). Left: schematic of the reporter constructs. Right: steady-state RNA levels normalized to *MYC* exon 1-reporter and mCherry internal control. (H) RNA stability and decay rates of *PVT1* exon 1 mutant reporters in HEK293T cells (*n* = 3). Left: RNA stability measurement. Right: RNA decay rates. (I) RNA levels of *PVT1* exon 1 mutant reporters after CHX and ActD treatment in COLO320DM (*n* = 3). RNA abundance normalized to DMSO and mCherry internal control RNA. (D, E, and G–I) RNA levels were measured by RT-qPCR, *p* > 0.03 (ns), *p < 0.03, **p < 0.002, ***p < 0.0002, ****p < 0.0001 by two-tailed *t* test. (E and G–I) Data are represented as mean ± SEM. See also Figure S4 and Tables S3 and S4.

To investigate the molecular mechanism underlying *PVT1* exon 1-mediated RNA stabilization, we explored the protein interactome of *PVT1* exon 1 and *MYC* RNA, as RNA stabilization often involves RNA-binding proteins (RBPs).^45^ We employed ChIRP-MS (comprehensive identification of RNA-binding proteins by mass spectrometry), a technique that identifies the RNA-associated proteome by pulldown of RNA of interest^46^ (Figure 4A). Given that *PVT1-MYC* accounts for 82% of total *MYC* transcripts in COLO320DM, while canonical *MYC* dominates (87%) in COLO320HSR (Figure S3B), we performed comparative ChIRP-MS against *PVT1* exon 1 and canonical *MYC* exons 1–3 to capture total *MYC* transcripts in both cell lines, along with RNase-treated controls to remove RNA-independent backgrounds. We also performed ChIRP-MS against *PVT1* exon 1—the common *PVT1* fusion region—in COLO320DM.

We identified 69 proteins enriched in *PVT1* exon 1 or *MYC* ChIRP-MS in COLO320DM, relative to *MYC* ChIRP-MS in COLO320HSR, suggesting preferential binding to *PVT1-MYC* transcripts (Figure 4B; Table S4). These proteins were RBPs predominantly involved in cytoplasmic translation, mRNA processing, and stability regulation (Figure 4B), implicating translation-coupled RNA stability control. *PVT1-MYC* replaces the *MYC* exon 1 5′ untranslated region (UTR),^47^ a regulatory platform for translational controls,^48^ with *PVT1* exon 1 while preserving the intact *MYC* open reading frame (ORF) from exon 2 (Figure 4C). As canonical *MYC* mRNA stability is known to increase upon translation inhibition,^49^ we tested whether *PVT1* fusion alters this response. Indeed, blocking translation with cycloheximide (CHX) stabilized canonical *MYC* but not *PVT1-MYC* RNAs (Figures 4D and S4A). Similarly, inhibiting nonsense-mediated decay (NMD)—a translation-coupled decay pathway—with an SMG1 inhibitor increased canonical *MYC* but not *PVT1-MYC* mRNA (Figure S4B), indicating that *PVT1* exon 1 fusion enables escape from translation-coupled decay.

To identify the direct effector RBPs involved in *PVT1* exon 1-mediated RNA stabilization, we intersected three datasets: *PVT1-MYC* ChIRP-MS hits, RBPs with binding motifs in *PVT1* exon 1, and eCLIP (enhanced crosslinking and immunoprecipitation)^50^-defined RBPs enriched on *PVT1* exon 1 (Figure S4C; Table S4). This analysis highlighted two serine/arginine-rich splicing factors (SRSFs, SR proteins)—SRSF1 and SRSF7—known regulators of RNA metabolism.^51–54^ eCLIP data showed strong and consistent enrichment of SRSF1 on *PVT1* exon 1 across multiple cell lines, but minimal occupancy of SRSF7 (Figure S4D). Together with SRSF1’s known function in RNA stabilization^52^ and NMD regulation,^55^ SRSF1 emerged as a strong candidate effector. Supporting this, RNA immunoprecipitation (RNA-IP) of SRSF1 in COLO320DM revealed significantly higher SRSF1 occupancy on endogenous *PVT1-MYC* than canonical *MYC* mRNAs (Figures 4E and S4E).

To examine the role of SRSF1 binding in *PVT1* exon 1-mediated RNA stabilization, we first characterized the *PVT1* exon 1-SRSF1 interaction. *De novo* structure prediction with AlphaFold3^56^ revealed that two RNA recognition motifs (RRMs) of SRSF1 interact with the 5′ end of *PVT1* exon 1 containing the known SRSF1 binding motif (GAGGA) (Figures 4F and S4F). To disrupt this interaction, we performed scanning mutagenesis of the *PVT1* exon 1-fused *MYC* reporter, introducing point mutations (GGA to UUA) in three putative SRSF1 binding sites while avoiding promiscuous effects from global perturbation of SRSF1’s role as a splicing factor and common essential gene.^57^ DeepCLIP^58^—a deep learning model trained on Encyclopedia of DNA Elements (ENCODE) SRSF1 eCLIP data—showed reduced SRSF1 binding scores at the mutated sites (Figure S4F). In parallel, we generated 75-nt deletion mutants spanning *PVT1* exon 1 to define the minimal sequence responsible for stabilization (Figure 4G).

Reporter assays in ecDNA-negative HEK293T cells demonstrated that the *PVT1* exon 1-fused reporter exhibited increased RNA levels (Figure 4G), consistent with the results in COLO320DM cells (Figures S3C and S3F), indicating a general stabilizing effect across cell types. Deletion of the AlphaFold3-predicted SRSF1 binding site (Del1 and Del2) markedly reduced RNA levels, with removal of the 5′ 75 nt (Del1) completely abolishing RNA upregulation and stabilization, reducing it to canonical *MYC* reporter levels (Figures 4G and 4H). Similarly, point mutations in the SRSF1 binding sites (pMut) significantly decreased RNA levels and stability (Figures 4G and 4H). RNA-IP confirmed reduced SRSF1 binding to these mutants (Figure S4G), supporting a role of SRSF1 interaction in *PVT1-MYC* RNA stabilization.

To test whether the 5′ 75 nt of *PVT1* exon 1 and SRSF1 binding are required for *PVT1* exon 1-mediated escape from translation-coupled decay, we expressed reporters and measured RNA stability under concurrent inhibition of translation and transcription. Consistent with the endogenous results (Figure 4D), the wild-type *PVT1*-*MYC* reporter RNA stability remained resistant to CHX, whereas the canonical *MYC* reporter was stabilized (Figure 4I). By contrast, *PVT1* exon 1 mutants regained CHX sensitivity (Figure 4I), consistent with their reduced RNA stability and SRSF1 binding (Figures 4H and S4G). These results indicate that SRSF1 binding to the 5′ end of *PVT1* exon 1 is important for evading translation-coupled decay. Supporting its functional significance, the 5′ end of *PVT1* exon 1 is highly conserved between mouse and human,^32,35^ despite lncRNA generally being poorly conserved.^59,60^ Consistent with this, long-read data revealed no RNA breakpoints in this region (Figure S4H), suggesting selective pressure to preserve it intact on ecDNA.

### *PVT1* fusion enhances the oncogenic function of *MYC*

**Figure 5.**
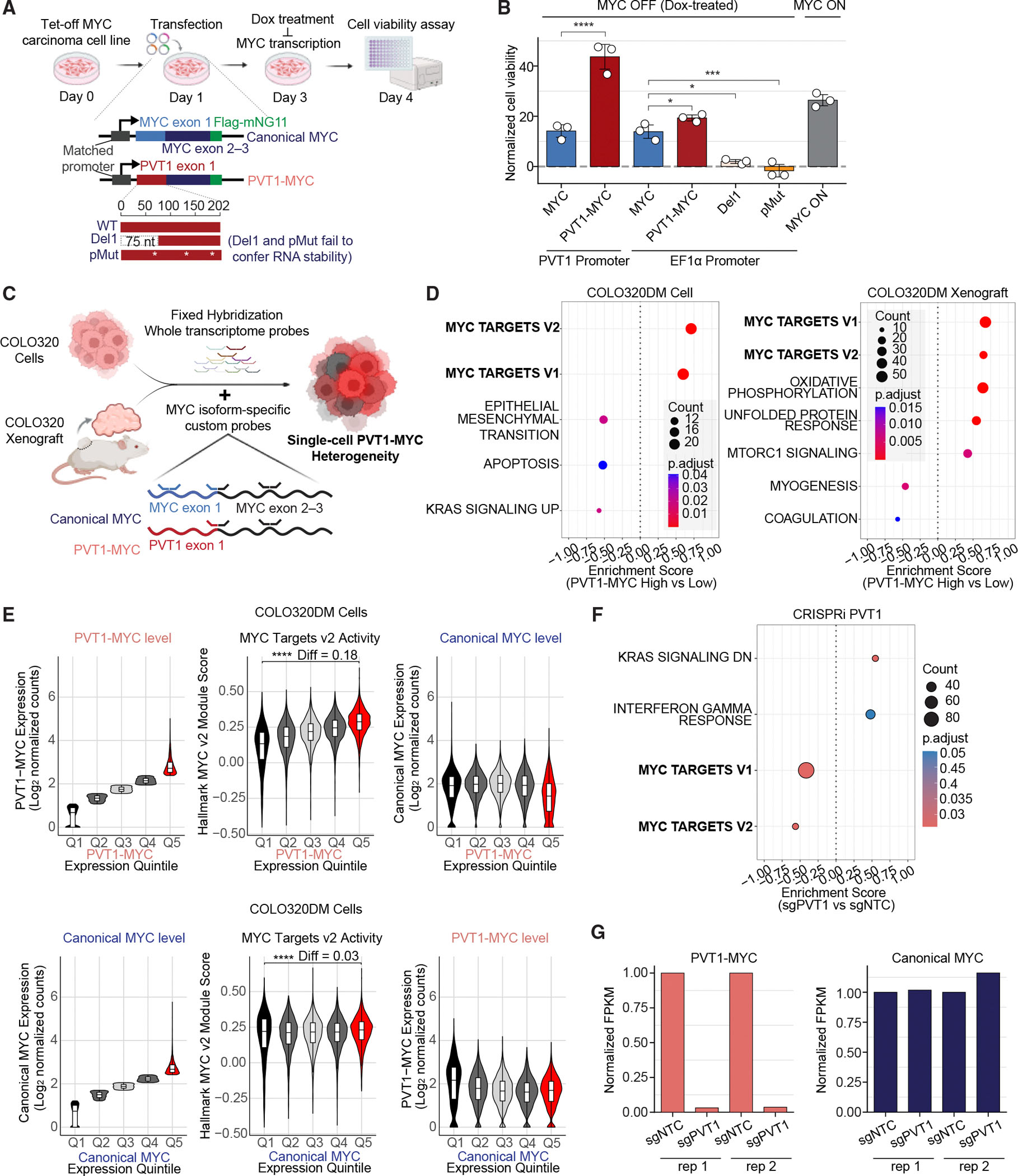
*PVT1* fusion enhances the oncogenic function of *MYC* (A) Schematic of the *MYC* rescue experiment in Tet-off *MYC* carcinoma cell line (EC4). (B) Normalized cell viability relative to the *GFP* negative control in the *MYC* rescue experiment (*n* = 3). Data are represented as mean ± SEM (*p < 0.03, ***p < 0.0002, ****p < 0.0001; one-way ANOVA test). (C) Schematic of Flex scRNA-seq in COLO320DM and COLO320HSR cells and xenograft models. (D) Hallmark pathways enriched in Q5 versus Q1 of *PVT1-MYC* cells from Flex scRNA-seq of COLO320DM cells and the xenograft model. (E) Violin plots displaying MYC targets v2 activity and expression level of *MYC* isoforms across expression quintiles of *PVT1-MYC* (top) and canonical *MYC* (bottom) in COLO320DM cells (****p < 0.0001; Wilcoxon rank-sum test). (F) Hallmark pathways enriched in CRISPRi sgPVT1 versus sgNTC (non-targeting control) RNA-seq of COLO320DM cells. (G) Normalized RNA expression levels of *PVT1-MYC* and canonical *MYC* in CRISPRi sgPVT1 RNA-seq of COLO320DM cells, relative to sgNTC. See also Figure S5 and Tables S3 and S5.

To investigate how *PVT1-MYC* functions in cancer cells, we examined the ability of *PVT1-MYC* to substitute for the oncogenic function of *MYC* in *MYC*-addicted cancer cells.^61–64^ *MYC*-addicted EC4 cancer cells were engineered to deplete canonical MYC upon doxycycline treatment, leading to severe cell death within 24 h.^65^ We transfected equal amounts of DNA constructs encoding either canonical *MYC* or *PVT1-MYC* along with mutants 24 h prior to MYC depletion and measured cell viability 24 h after endogenous MYC depletion (Figure 5A). We found that *PVT1-MYC* more efficiently rescues cancer cell viability in response to MYC depletion compared with canonical *MYC*, and combination with the *PVT1* promoter further increased cell viability (Figure 5B). Furthermore, a 75-nt deletion at the 5′ end (Del1) or point mutations (pMut) of *PVT1* exon 1 abrogated the ability of *PVT1-MYC* to rescue MYC silencing. These results demonstrate that the 5′ 75 nt of *PVT1* exon 1, containing SRSF1 binding site, is critical for *PVT1-MYC* to rescue MYC oncogenic activity.

As the *MYC* oncogene encodes a transcription factor that regulates downstream gene expression, we next investigated whether the *PVT1-MYC* can regulate the expression of MYC target genes. Using Flex single-cell RNA-seq (scRNA-seq),^66,67^ a whole-transcriptome profiling method that allows detection of transcript isoforms in single cells via isoform-specific probes (Table S3), we measured *MYC* isoform-specific RNA expression of COLO320DM and COLO320HSR single cells both *in vitro* and *in vivo* xenografts (Figure 5C). Due to a lack of centromeres, ecDNAs are randomly inherited during cell division, leading to copy-number heterogeneity in the cell population.^8,9^ Leveraging the cell-to-cell variation detection for *PVT1-MYC* RNA expression in COLO320DM (Figure S5A), we examined the pathways regulated by *PVT1-MYC* expression by binning cells based on their expression.

Hallmark gene sets enrichment analysis revealed that cells with high *PVT1-MYC* expression (Q5) showed significantly upregulated MYC targets compared with cells with low *PVT1-MYC* expression (Q1), both *in vitro* and *in vivo* (Figures 5D and S5B; Table S5). Additionally, MYC target scores showed a stronger dose-dependent increase with *PVT1-MYC* expression than with canonical *MYC* levels both *in vitro* and *in vivo* (Figures 5E and S5C), indicating that *PVT1-MYC* more potently activates MYC targets. In COLO320HSR cells, where *PVT1-MYC* expression is low, canonical *MYC* correlated more strongly with MYC target scores than in COLO320DM cells (Figure S5D). These results suggest that when highly expressed, *PVT1-MYC* dominates the oncogenic function of *MYC*. Supporting this, CRISPR interference (CRISPRi) of the *PVT1* promoter in COLO320DM cells—selectively reducing *PVT1-MYC* but not canonical *MYC*—decreased MYC target genes (Figures 5F and 5G; Table S5).

**Figure 6.**
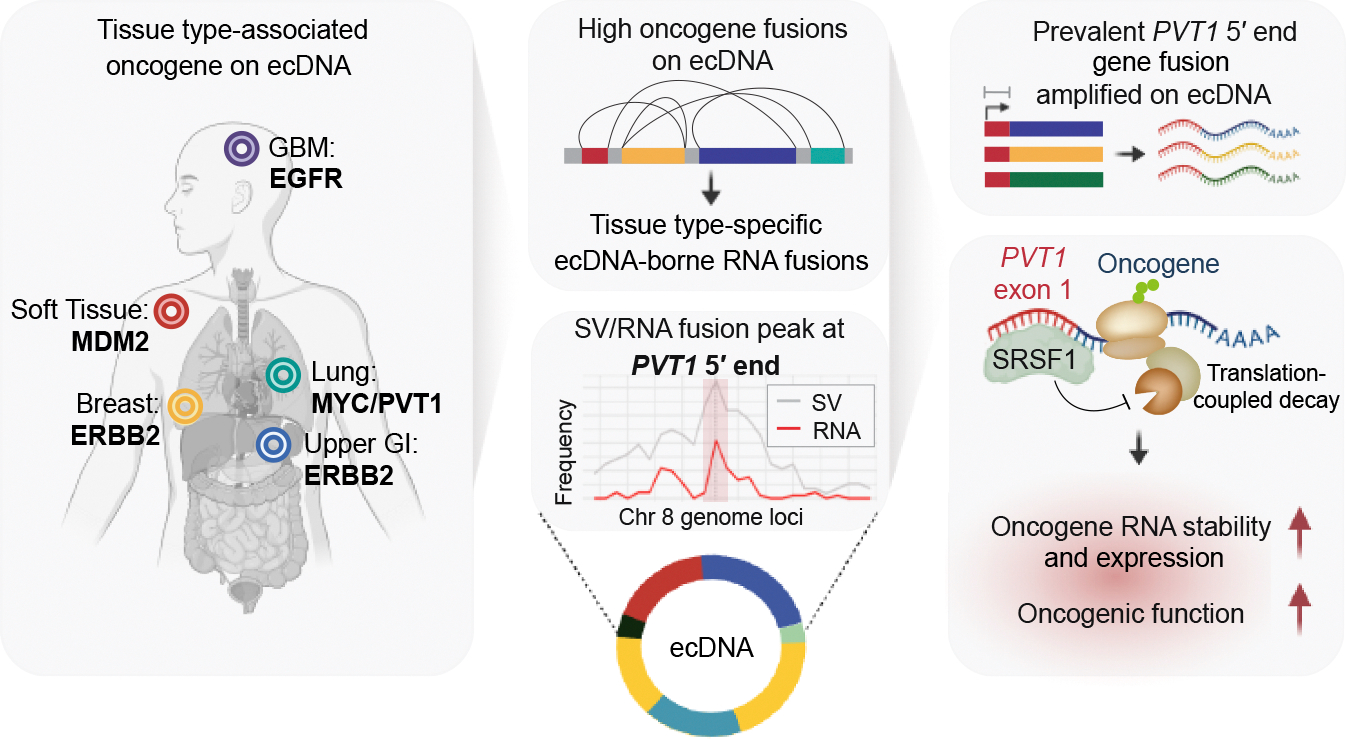
Model for ecDNA-borne oncogene RNA fusions EcDNA is a key driver of oncogene fusions and amplifies oncogenic fusion transcripts in a cancer type-specific manner. *PVT1* with exon 1 is the most prevalent ecDNA-borne fusion partner, which enhances RNA stability through SRSF1 binding that enables escape from translation-coupled decay and potentiates oncogenic function. This model proposes ecDNA as a platform for generating and selecting functional rearrangements in cancer.

Beyond MYC targets, Gene Ontology (GO) analysis comparing cells with high versus low *PVT1-MYC* expression revealed enrichment for protein synthesis-related pathways that were previously linked to MYC activation^68^ (Figure S5E). By contrast, the low *PVT1-MYC* group was enriched for pathways such as “negative regulation of Wnt signaling,” suggesting reduced stemness and proliferative capacity.^69^ Together, our data demonstrate that *PVT1-MYC* enhances *MYC* oncogenic activity, conferring a more potent role in cancer cell survival and gene regulation that likely underlies its selection as the most enriched oncogene fusion on ecDNA in cancer (Figure 6).

## DISCUSSION

### EcDNAs are platforms for gene fusion

EcDNAs are pernicious drivers of tumor evolution, acting as platforms for massive oncogene expression and rapid genome alterations. Dynamic genome alteration and uneven inheritance of ecDNAs generate intratumoral genetic heterogeneity that, under selective pressure, reinforces tumor fitness.^3,9,10^ The strong association between *PVT1* fusion and ecDNA likely reflects both structural fragility and selective advantage. *PVT1*, a known breakpoint hotspot in Burkitt’s lymphoma,^31,32^ may undergo increased fragmentation and reassembly on ecDNA, where DNA damage response and repair activity are elevated.^70,71^ Particularly, in the presence of multiple ecDNA copies in ecDNA hubs,^4^ combination of structural fragility of *PVT1* with ecDNA may facilitate iterative fusion events. Supporting this, in COLO320DM ecDNA, *PVT1-MYC* fusions exist in multiple copies with defined orientation,^4,72^ suggesting multiple fragmentation and reassembly events rather than a single and random genomic rearrangement. Once formed, *PVT1* fusions may be selectively retained on ecDNA, similar to the preferential maintenance of constitutively active EGFRvIII mutants on ecDNA in glioblastoma,^10^ reinforcing the link between oncogenic genetic variations and ecDNA.

Our study highlights the clinical potential of ecDNA-borne oncogene fusion RNAs as biomarkers for detecting ecDNA-positive cancers. EcDNA can arise in the precancerous stage and expand its structural complexity and copy number during progression,^12^ underscoring an urgent need for sensitive detection strategies. We show that ecDNA-borne RNA fusions exhibit elevated RNA expression and tissue-type specificity, and that combining *PVT1* fusion RNA detection with copy-number analysis improves ecDNA detection accuracy. Beyond *PVT1*, our comprehensive catalog of ecDNA-borne RNA fusions and their prevalence across cancers offers a resource for novel diagnostics. Moreover, ecDNA-borne oncogene fusions can generate tumor-specific neoantigens, presenting promising opportunities for mRNA vaccine-based therapy.^73,74^ As these fusions are absent in normal cells yet amplified via ecDNA in cancers, they present precise targets for detection and treatment of ecDNA-driven cancers. Future studies dissecting their pathogenic roles will provide mechanistic insights into their selection on ecDNA and inform therapeutic strategies for targeting ecDNA-driven oncogenesis.

### Oncogene activation by *PVT1* 5′ fusions

This study highlights an unexpected consequence of gene fusion on post-transcriptional RNA regulation. Gene fusions in cancer have previously been considered in the context of altered transcriptional regulation (e.g., *TMPRSS2-ERG*)^75–77^ or altered protein products (BCR-ABL).^78,79^ Recognition of their roles in cancer has led to improved cancer diagnostic strategies and targeted therapies. Our studies of *PVT1* 5′ fusions indicate that *PVT1* exon 1 serves as a universal element that stabilizes diverse partner RNAs, including *MYC*, *CASC8*, and *MYH7*. For short-lived oncogenic mRNAs such as *MYC*, *PVT1* boost could be critical for extending oncogene mRNA function. Our data indicate an SRSF1 motif-dependent mechanism for *PVT1*-mediated mRNA stabilization. We recognize the possibility that additional factors may function through these motifs. Exploring other contributors to *PVT1* exon 1-mediated regulation will be worth investigating in future studies.

In addition to RNA stabilization, prior studies suggest that *PVT1* fusion may also rewire enhancer-promoter regulation. Our previous studies found that the promoter of *PVT1* and *MYC* competes for enhancer binding,^35^ and *MYC* expression can negatively regulate its own transcription in normal cells.^35,80^ Cancer cells may exploit *PVT1* 5′ end fusions, replacing the *MYC* promoter with the *PVT1* promoter, thereby evading the negative feedback loop.^35^ Additionally, the *PVT1* promoter also facilitates episomes to join ecDNA hubs—nuclear clusters of ecDNAs that boost transcription.^4^ Therefore, *PVT1* 5′ end fusion on ecDNA may not only circumvent autoregulatory feedback but also synergistically enhance *PVT1-MYC* expression through both *PVT1* exon 1-mediated RNA stabilization and rewired *cis* and *trans*-transcriptional activation within ecDNA hubs.

In summary, our study uncovers ecDNA as a major platform for generating oncogenic fusion transcripts. Future studies dissecting the mechanisms underlying how ecDNA-enriched oncogene fusions drive tumor progression or treatment resistance will be key to developing clinically actionable strategies targeting ecDNA-positive cancers.

### Limitations of the study

Our analysis of ecDNA-borne gene fusions is potentially influenced by the limited sample numbers in certain cancer types. In this study, we focused on the fusion transcripts resulting from genomic rearrangements, as 88.5% of ecDNA-borne RNA fusions were supported by SVs. Nonetheless, fusion transcripts can also arise through genomic rearrangement-independent mechanisms such as *trans*-splicing or alternative splicing of readthrough transcripts.^15,81^ While we establish a critical role for *PVT1* 5′ fusions in RNA stabilization and oncogenic activation, the specific roles of SRSF1 binding and other RBPs, as well as the potential interplay of additional mechanisms—transcription, translation, oncoprotein isoforms, and cytoplasmic decay—in *PVT1* fusion-mediated oncogenic function, remain to be defined. Further investigation into the roles of additional ecDNA-borne SVs will be important for deepening our understanding of the molecular mechanisms underlying ecDNA-driven cancers.

## STAR★METHODS

### RESOURCE AVAILABILITY

#### Lead contact

Requests for further information and resources should be directed to and will be fulfilled by the lead contact, Howard Y. Chang (howchang@stanford.edu).

#### Materials availability

This study did not generate new, unique reagents.

#### Data and code availability

All software, sequencing data from this study, and the sources of publicly available data used in this study are listed in the key resources table. Raw and processed sequencing data of Flex scRNA-seq, CRISPRi PVT1 RNA-seq, long-read RNA, and DNA sequencing have been deposited at NCBI BioProject accession: PRJNA1346268. Custom code is available at GitHub at https://github.com/ShuZhang0917/ecDNA-borne_fusion.git. Source imaging data have been deposited at the Stanford Digital Repository (https://doi.org/10.25740/cp783mb3217), and original western blot images have been deposited at Mendeley Data: https://www.doi.org/10.17632/cnz75cv3cs.2. Any additional information required to reanalyze the data is available from the lead contact upon request.

### EXPERIMENTAL MODEL AND STUDY PARTICIPANT DETAILS

#### Cell culture

The parental COLO320DM and PC3 lines were obtained from ATCC. COLO320DM and PC3DM lines were isolated by the Mischel Lab through single-cell expansions of the parental lines.^3,91^ The GBM39HSR and GBM39KT cell lines are patient-derived neuro-sphere cell lines and were established as previously described.^3,10,92^ All the other cell lines were purchased from ATCC. Human colorectal cancer cell line COLO320DM, COLO320HSR, and HCT116; prostate cancer cell line PC3DM; gastric cancer cell line SNU16; and embryonic kidney cell line HEK293T were cultured in 4.5 g l^−1^ glucose-formulated Dulbecco’s Modified Eagle’s Medium (DMEM; Thermo Fisher Scientific, 11995073) supplemented with 10% fetal bovine serum (FBS; Gibco) and 1% penicillin-streptomycin (pen-strep; Thermo Fisher Scientific, 15140163). Lung cancer cell lines (NCI-H82, NCI-H2170, MSTO-211H, and NCI-H524) were cultured in ATCC-formulated RPMI-1640 medium (ATCC 30–2001) supplemented with 10% FBS and 1% pen-strep. GBM39 cell lines were cultured in Dulbecco’s Modified Eagle’s Medium/F12 (Gibco, 11320–033) supplemented with 1× B27 (Gibco, 17504–01), 20 ng ml^−1^ epidermal growth factor (Sigma, E9644), 20 ng ml^−1^ fibroblast growth factor (Peprotech, AF-100–18B), 1–5 μg ml^−1^ heparin (Sigma, H3149), 1× GlutaMAX (Gibco, 35050–061) and 1% pen-strep. All the cells were maintained at 37 °C with 5% CO2 in a humidified incubator. Cell lines routinely tested negative for mycoplasma contamination.

#### Generation of xenograft mice model

Female athymic nude mice (Charles River Laboratories) were housed under standard conditions and used for subcutaneous tumor implantation. Prior to injection, mice were anesthetized using isoflurane in an induction chamber. A 100 μL aliquot of the prepared cell suspension was injected bilaterally into the subcutaneous space of the flanks using a 25-gauge needle and a 1 mL syringe. Mice were monitored post-procedure for signs of distress or complications.

At the experimental endpoint, mice were euthanized using a sealed CO_2_ chamber, with cervical dislocation performed as a secondary confirmatory method. Tumors were sharply dissected from the subcutaneous space, immediately flash-frozen in liquid nitrogen, and stored at −80°C for future analysis.

### METHOD DETAILS

#### RNA Fusion analysis using TCGA and CCLE databases

We applied a comprehensive filtering strategy for unfiltered RNA fusions identified by STAR-Fusion within the TCGA and CCLE RNA-seq datasets, details as described below. We first collected the unfiltered RNA fusion data from CCLE with 1,482 cell lines, sourced from the DepMap 24Q2 Public dataset (OmicsFusionUnfilteredProfile.csv), and from TCGA encompassing 9,426 tumor samples and 707 normal samples, extracted from Table S2 of Haas et al.^25^ To minimize potential false positives, we implemented several exclusion criteria based on previous studies^19,25,93^: (1) Red Herrings: Fusion transcripts classified as Red Herrings by FusionInspector (version 2.8.0) were excluded based on known fusions derived from normal samples, as specified in GRCh38_gencode_v37_CTAT_lib_Mar012021.plug-n-play.tar.gz. (2) Gene Origin: Fusion partners that originated from the same gene or included paralogous genes, as annotated in the GRCh38.p14 GTF file and accessed through Ensembl Biomart, were discarded. (3) Normal Fusions: We filtered out common fusion transcripts identified in cancer samples that were recurrently observed in TCGA normal samples. (4) Gene Types: Fusion partners involving mitochondrial genes, HLA genes, or immunoglobulin genes were also removed from our analysis. (5) Expression Thresholds: Non-recurrent fusions with low expression levels, defined as a Fusion Fragments Per Million (FFPM) < 0.1, were excluded. Additionally, recurrent fusions with a maximum FFPM less than 0.05 were filtered out. Furthermore, we removed highly recurrent fusions that were exclusive to CCLE, characterized by the same breakpoints in at least 10 samples, as long as they were not present in TCGA tumor samples.

#### Amplicon type analysis

AmpliconArchitect (AA) was utilized to identify focally amplified regions, which were classified into four distinct types: extrachromosomal DNA (ecDNA), breakage-fusion-bridge (BFB) cycles, complex non-cyclic amplifications, and linear amplifications. Genomic regions without any detected focal amplifications were defined as no focal somatic CN amplification (No-fSCNA^5^). The AmpliconArchitect results were obtained from the repository at https://ampliconrepository.org/,^24^ where we downloaded data for 329 CCLE cell lines and 2,471 TCGA samples.

For the structural variants (SVs), we retrieved CCLE SV data for 329 cell lines from DepMap (CCLE_translocations_SvABA_20181221).^94^ TCGA somatic SVs from 844 tumor samples that overlapped with AmpliconArchitect samples were acquired from the GDC (Genomic Data Commons) data portal at https://portal.gdc.cancer.gov/. Both CCLE and TCGA SVs were identified using the SvABA tool.

To ensure the integration of amplicon types, SVs, and RNA fusions that were originally aligned to different versions of the human genome, we converted the TCGA AmpliconArchitect results and CCLE SVs from GRCh37 to GRCh38 using the Liftover tool.^95^ Then, we aligned TCGA Tissue Source Site and the participant number to match RNA-seq data to the WGS data from the same patient. Subsequently, we annotated the genes associated with the focal amplicons as well as the nearest genes within a 100 kb range, both 5’ and 3’ of the SV breakpoints, utilizing the GRCh38.p14 GTF file. To identify SVs supporting gene fusions (SVGF) producing fusion transcripts, SV breakpoints were assigned if they were less than 500 kb from RNA fusion breakpoints and within 100 kb of a gene body.^14^ Finally, if the RNA fusion breakpoints and SV breakpoints were located within amplicons associated with specific amplicon types, we categorized those breakpoints accordingly. This classification allowed us to associate each breakpoint with its respective amplicon type, facilitating further analysis to compare the features of SVs and RNA fusions from different amplicon types.

#### Analysis of fusion rate and burden for SVs and RNA fusions

Using the matched samples with AA, SV and RNA fusion data, the rate for SVGF in each amplicon interval was calculated as dividing the number of genes with SV breakpoints within or near (< 100 kb) the gene body,^26–28^ by the total number of genes within each amplicon interval. The RNA fusion rate was determined as the number of gene with RNA fusion breakpoints within the gene body divided by the total number of gene within each amplicon interval.

SV burden^13^ and RNA fusion burden were calculated as the proportion of cancer samples harboring SV or RNA fusion breakpoints within 100 kb genomic windows across entire genome. The number of samples with SV or RNA fusion breakpoints were divided by the total number of samples linked to each amplicon type.

#### Enrichment analysis of ecDNA-borne fusion oncogenes by cancer type

To evaluate whether an oncogene fusion is preferentially enriched on ecDNA, we compared the observed versus expected proportions of samples harboring ecDNA-borne versus non-ecDNA-borne fusions (Figure 1D). The expected proportions were derived under a null model assuming a random distribution of RNA fusions, weighted by the overall frequency of ecDNA-borne and non-ecDNA-borne fusions across all samples.

To evaluate the enrichment of each ecDNA-borne RNA fusion oncogene in each cancer type, we performed a one-tailed hypergeometric test using the phyper function in R. This approach calculates the probability of observing more ecDNA-borne fusion-positive samples by chance, assuming fusion events are distributed independently of ecDNA amplification status. A significant *p*-value (< 0.05) indicates an enrichment of fusion events occurring on ecDNA in that cancer type. Only oncogenes with significant enrichment (*p*-value < 0.05) are shown in the heatmap of Figure 1F. Cancer types that have ≥ 5 samples with ecDNA oncogene amplification and at least one sample with RNA fusions are included in Figure 1F.

For each gene-cancer type pair, the test estimates the probability of observing the actual number of ecDNA-borne fusion events or more, under the null hypothesis of random association between RNA fusion and ecDNA amplification. Parameters of (phyper(q - 1, m, n, k, lower.tail = FALSE)) were defined as follows:

q: number of samples with RNA fusion involving an ecDNA-amplified gene;

m: total number of samples with the RNA fusion (regardless of ecDNA status);

n: number of samples without the RNA fusion;

k: number of samples with amplification of the gene on ecDNA.

#### Long-read RNA sequencing

Total RNA was extracted by Direct-zol^™^ RNA Purification miniprep Kit (Zymo Research, R2052). RNA integrity (RIN) was confirmed by Bioanalyzer and RNA samples with maximum RNA integrity (RIN = 10) were used for Nanopore library preparation. We constructed Nanopore libraries using the Oxford Nanopore Direct RNA Sequencing Ligation Kit (SQK-RNA002) according to manufacturer’s instructions. We sequenced libraries on an Oxford Nanopore PromethION using a R.9.4 Flow Cell (FLO-PRO002) according to manufacturer’s instructions.

To identify fusion transcripts, we utilized Jaffa^96^ (version 2.3) on our Oxford Nanopore Direct RNA-sequencing (DRS) data. Specifically, basecalls were derived from raw FAST5 signal using Guppy (using the 2020–09-21_rna_r9.4.1_promethion_256_855130ab RNA basecalling model). Then, Jaffa was invoked using the default .groovy pipeline with the following flags: -n 20. The resulting Jaffa file was used to determine the support and confidence of each identified fusion transcript.

#### Long-read DNA sequencing library preparation

High-molecular weight (HMW) genomic DNA of COLO320DM, COLO320HSR, PC3DM and SNU16 cells was extracted from approximately 2 million using MagAttract HMW DNA kit (Qiagen, 67563). After extracting HMW gDNA, we constructed Nanopore libraries using the Oxford Nanopore Ligation Sequencing DNA kit V14 (SQK-LSK114) according to manufacturer’s instructions. We sequenced libraries on an Oxford Nanopore PromethION using a 10.4.1. Flow Cell (FLO-PRO114M) according to manufacturer’s instructions. Basecalls from raw POD5 files were computed using Dorado (v.0.7.2). DNA reads were aligned to hg38 using minimap2 (v2.26).

To detect SVs that support RNA fusion breakpoints, we considered SV breakpoints within or near (< 100 kb) the gene body.^26–28^ DNA long read that support the RNA gene fusions in Figure S1B were identified based on the SA tag of minimap2 aligned reads. *PVT1* DNA breakpoint positions in Figure S2I were identified using Sniffles2 (v2.0.7). ecDNA structure was reconstructed by referring to Complete Reconstruction of Amplifications with Long reads (CoRAL) for reconstructing ecDNA architectures using long-read data.^72^

#### Fixed scRNA-seq (Flex-seq)

Xenografts were dissociated according to 10x Genomics Tissue Fixation and Dissociation Demonstrated Protocol #CG000553 (Rev B). Xenografts were stored in liquid nitrogen until fixation where they were thawed at room temperature and sectioned into ~25 mg tissue samples which were titurated with a wide-bore P1000 in fixation buffer incubated for 20 hr at 4 °C. Fixed tissue sections were quenched and supplemented with Enhancer to 10% and 50% w/v Glycerol to 10% and stored at −80 °C. Frozen fixed tissues were washed and resuspended in 0.2 mg/ml Liberase (Millipore Sigma 5401020001) prewarmed to 37 °C and incubated for 30 minutes, samples were titurated and dissociation was evaluated by Countess II FL Automated Cell Counter (Thermo Fisher Scientific) every 10 minutes.

Cell samples were fixed with Chromium Next GEM Single-Cell Fixed RNA Sample preparation kit (10x Genomics 1000414) was used according to the manufacturer’s protocol. Aliquots of 2 million cells were fixed according to the Fixation of Cells and Nuclei Demonstrated Protocol # CG000478 (Rev D). Fixation for 20 hr at 4 °C, was quenched and supplemented with Enhancer to 10% and 50% w/v Glycerol to 10% and stored at −80 °C. Cryopreserved fixed samples were thawed and ~500,000 cells were hybridized using Chromium Fixed RNA Kit, Human Transcriptome, 4rxn × 4BC (PN-1000475, 10x Genomics). LHS and RHS custom probe pools were added to hybridization reactions according to their barcode to a final concentration of 2 nM following 10x Genomics Demonstrated Protocol #CG000621 (Rev D). Hybridized cell and xenograft samples were pooled and washed following the Pooled Wash Workflow #CG000527. Two lanes of GEMs were generated on the Chromium X (10x Genomics) targeting 60,000 cells recovered.

Single-cell gene expression of COLO320DM and COLO320HSR cells and xenografts was assayed using 10x Chromium Fixed RNA-Profiling scRNA-seq. Indexed libraries were quantified by Qubit dsDNA Quantitation, High-Sensitivity kit (Thermo Fisher Q32851) for yield and High-Sensitivity D1000 TapeStation (Agilent 5067–5585) to ensure expected library size and absence of contaminating products. Pooled libraries were sequenced by paired-end: Read 1: 28, I1: 10, I2: 10, Read 2: 90 (Illumina NovaSeq X).

#### scRNA-seq analysis

BCL Convert Software (Illumina) was used to generate demultiplexed FASTQ sequencing libraries which were aligned with Cellranger multi (v.8.0.0, 10x Genomics) using a human probeset reference (v1.0.1-GRCh38–2020-A) modified to include custom probe sequence references (Sequences provided in Table S3). Seurat objects were constructed from the Cellranger generated filtered feature-barcode matrices and further filtered for cells with greater than 200 detected genes and less than 10% of mitochondrial reads.^97^ Dimensionality reduction was performed on the top 2,000 most variable genes of log-normalized and scaled samples.

Expression quantiles for *PVT1-MYC* were generated by binning each sample by depth-normalized counts. *MYC* isoform driven differentially expressed genes were calculated on the output FindMarkers with a Wilcoxon rank-sum test comparing *PVT1-MYC* high (Q5) vs low (Q1) with a log2-foldchange threshold of 0.25. Gene-set enrichment on these differentially expressed genes was calculated by GSEA for Hallmark signatures. Cells were scored for the expression of Hallmark MYC v2 signature using the AddModuleScore function (Seurat) with 100 control genes. MYC v2 Hallmark module scores between *PVT1-MYC* quintiles were calculated by the Wilcoxon rank-sum-test. The difference (Diff) was calculated as the mean module score of Q5 subtracted by that of Q1 in Figures 5E, S5C, and S5D. Gene ontology (GO) enrichment analysis of significantly differentially expressed genes (Bonferroni correction-adjusted p-value < 0.01 and average log2 fold change > 0.5) was performed using Metascape.^88^ GO terms shared between COLO320DM cells and xenografts were considered to represent pathways regulated by *PVT1-MYC* fusion.

#### DNA FISH staining and imaging analysis

COLO320DM and COLO320HSR cells were arrested in metaphase with 100ng ml^−1^ KaryoMAX^™^ Colcemid^™^ Solution in PBS (Gibco) for 4 hr. The cells were collected after trypsinization and washed once in PBS. The cells were resuspended in 0.075M KCl for a 20-min incubation at 37°C, followed by fixation with Carnoy’s fixative (3:1 methanol: acetic acid). The cell pellet was washed thrice with Carnoy’s fixative prior to being dropped onto a humidified glass slide. After the sample was fully air-dried, the slide was briefly incubated in 2X SSC buffer, followed by dehydration in ascending ethanol concentrations of 70%, 85% and 100% for 2 mins each. FISH probes obtained from Empire Genomics were freshly diluted in hybridization buffer at 1:6 ratio. The *MYC* region was probed with RP11–440N18 (Chr 8:128,596,756 – 128,777,986 - hg19). and *PVT1* exon 1 region was targeted with the WI2 clone G248P89481C4 (Chr8:128,790,936–128,830,829 - hg19). The diluted probes were then added to the sample with a coverslip applied on top. The slide was subjected to heat denaturation at 75°C for 3 mins, followed by overnight incubation at 37°C for hybridization in a humidified slide moat. The next day, the coverslip was gently removed from the sample, and the slide was washed in 0.4X SSC and 2X SSC-0.1% Tween-20 for 2 mins each. DNA was stained with DAPI stain (50ng mL^−1^), followed by a brief wash in ddH_2_O and was left to air-dry. The sample was mounted with ProLong Diamond (Invitrogen). Image acquisition was performed on a Leica DMi8 widefield microscope with a 63x oil objective.

#### RNA stability assay

For endogenous mRNA stability assay, 400,000 of COLO320DM and COLO320HSR cells were seeded in 12 well plate 1 day before the actinomycin D treatment. Next day, cells were treated with 5 μg/mL of actinomycin D and collected in a time course manner (0, 1, 2, and 4 hr). For reporter mRNA stability assay, 200,000 of COLO320DM cells were seeded in 12 well plate 1 day before plasmid transfection and cells were transfected with 1 μg of plasmid DNA using the Lipofectamine 3000 Transfection Reagent (Thermo Scientific, L3000008) according to manufacturer’s instructions. Cells were treated with 5 μg/mL of actinomycin D 2 days post-transfection and collected in a time course manner (0, 2, 4 hr). For *PVT1* exon 1 mutant reporter mRNA stability assays, 100,000 of HEK293T cells were seeded in 12 well plate 1 day before plasmid transfection and RNA stability assay was done as described above.

#### Modeling RNA stability

To model the life cycle of RNA transcripts (Furlan et al.^98^), we used an ordinary differential equation to represent the synthesis and decay of RNA:

(Equation 1) $$\frac{dT}{dt}=\alpha -\beta \cdot T$$

where T, the total number of RNA transcripts, changes over time t depending on the rates of synthesis (α) and decay (β). At steady state, the change in RNA transcripts over time is zero:

$$\frac{dT}{dt}=0$$

Therefore,

(Equation 2) $$T_{0}=\frac{\alpha }{\beta }$$

To calculate the RNA decay rate β, we analyzed quantitative reverse transcription PCR data for COLO320DM and COLO320HSR cells after treatment with actinomycin D at various timepoints. RNA abundance is normalized to 0 hr and *GAPDH* internal control RNA. The total RNA transcripts at time t drop according to the following equation:

(Equation 3) $$T(t)=T_{0}\cdot e^{-\beta \cdot t}$$

where total RNA T(t) at time t is a function of total RNA at the start of transcription block, T0, the RNA decay rate β, and time t. This gives the RNA decay rate

(Equation 4) $$\beta =\frac{ln\left(\frac{T_{0}}{T(t)}\right)}{t}$$

β was calculated for each transcript (canonical *MYC* and *PVT1-MYC*) for each experimental replicate in each cell line at each time point, and the mean β value of each replicate was calculated using all time points. Finally, to compare the contribution of RNA decay to total RNA transcript levels, we compared the RNA decay rates β of canonical *MYC* and *PVT1-MYC* with the total transcript levels per DNA copy at steady state using Equation 2.

#### Translation inhibition assay

400,000 of COLO320DM and COLO320HSR cells were seeded 1 day before cycloheximide (CHX) treatment in 12 well plate. Next day, cells were treated with CHX at final 10 μg/mL and incubated for 15 min at 37°C. Then cells were treated with actinomycin D (ActD) at final 5 μg/mL and harvested in a time course manner (0, 2, 4 hr). RNAs were extracted and RNA stability was measured by RT-qPCR. For reporter assays, 60,000 of COLO320DM cells were seeded in 24 well plate 1 day before plasmid transfection and cells were transfected with 100 ng of plasmid DNA using the Lipofectamine 3000 Transfection Reagent (Thermo Scientific, L3000008) according to manufacturer’s instructions. CHX and ActD were treated as described above, 48 hr post-transfection and cells were harvested at 4 hr of ActD treatment. RNAs were extracted and RNA stability was measured by RT-qPCR.

#### NMD inhibition assay

60,000 of COLO320DM cells were seeded 1 day before SMG1i treatment in 24 well plate. Next day, cells were treated with SMG1i at 0.5, 1, 2 μM and incubated for 6 hr at 37°C. RNAs were extracted and quantified by RT-qPCR. *PUMA* mRNA, a known NMD target was used for positive control.

#### Reporter Plasmid Construction

To generate reporter constructs for testing *PVT1* exon 1 fusion effect on RNA expression under the matched promoter–either *PVT1* or control promoters (Figure S3C), sequence of *PVT1* promoter (chr8:127793691–127794532, hg38), *PVT1* exon 1 (chr8:127794533–127794734, hg38) and minimal promoter was amplified from the plasmid constructs (*PVT1*p-nLuc and minp-nLuc) used in Hung et al.^4^ and subcloned to reporter plasmids driving NanoLuc luciferase (nLuc) and a constitutive thymidine kinase (TK) promoter driving Firefly luciferase (fLuc) as an internal control by Gibson assembly.

To generate *MYC*-Flag-mNG11 reporter for testing *PVT1* exon 1 fusion function under the matched promoter–either *PVT1* or control promoters (Figures 3F, S3F, 4G–4I, and S4G), endogenous *PVT1-MYC* and canonical *MYC* sequence was amplified from cDNA that was reverse transcribed from total RNA of COLO320DM using Primescript^™^ RT Reagent Kit with gDNA Eraser (Takara Bio, RR047A) according to manufacturer’s instruction, and subcloned to a vector with Flag-mNG11 tag and a constitutive thymidine kinase (TK) promoter driving mCherry as an internal control.

#### RT-qPCR

RNA was extracted using RNeasy Plus mini Kit (Qiagen, 74136) or Maxwell RSC simplyRNA Cells Kit (Promega, AS1390) with DNase treatment. 10 ng of RNA was used for RT-qPCR with 1× Brilliant II qRT-PCR mastermix with 200 nM forward and reverse primer and 0.5 μl RT/RNase block (Agilent, 600825) for 10 μl of total reaction. Each Ct value was measured using Lightcycler 480 (Roche) and each mean dCt was averaged from a duplicate RT-qPCR reaction with biological replicates. This list of RT-qPCR primers is shown in Table S3. Common forward primer against *PVT1* exon 1 was used across *PVT1* exon 1-fused RNAs.

#### ChIRP-MS

Cell harvesting, lysis, and ChIRP were performed largely as previously described by Chu et al.^99^ Approximately 100 million cells were fixed with 3% formaldehyde for 30 min, followed by final 125 mM Tris-HCL pH 8.0 (Invitrogen, 15568–025) quenching for 5 min. Lysate was generated by resuspending cell pellets in 1 mL lysis buffer (50 mM Tris-pH 7.0, 10 mM EDTA, 1% SDS) per 100 mg of cell pellet weight. Sonication was done using a focused-ultrasonicator (Covaris E220) until the RNA length was ~500 nt as determined by agarose gel analysis and stored at −80°C. Lysates were thawed on ice and precleared by using 30 μL washed MyOne C1 beads (Thermo Fisher Scientific, 65002) per mL of lysate at 37°C for 30 min on rotation. Preclearing beads were removed twice from lysate using magnetic stand. For RNase control ChIRP, precleared lysates were treated with 30 μg of RNase A (Fisher Scientific, 12–091-021) per mL of lysate and incubated at 37°C for 45 min. Next, every 1 mL of experimental and RNase control ChIRP lysates were incubated with 100 pmole of biotinylated probes targeting *PVT1* exon 1 and *MYC* exon 1–3 for total *MYC* and *PVT1* exon 1 for *PVT1*-fusion transcripts respectively, with 2 mL of hybridization buffer (750 mM NaCl, 1% SDS, 50 mM Tris-HCl pH 7.0, 1 mM EDTA, 15% formamide) and incubated at 37°C for 16 hr on rotation. ChIRP probe pools were composed of an equimolar mix of antisense oligos (see Table S3 for sequence). Next day, 100 μL of MyOne C1 beads for every 100 pmoles of probes (; per mL of lysate) were washed three times before use and incubated with lysates at 37°C for 45 min on rotation. RNA-protein interactome by ChIRP was collected on the beads with a magnetic stand and beads were washed 5 times for 2 min with constant mixing in 1 mL of ChIRP wash buffer (2x NaCl-Sodium Citrate (SSC, ThermoFisher Scientific), 0.5% SDS) at 37°C. At the last wash, 1% of beads were saved for RNA extraction for RNA pull-down QC. The enriched proteins were eluted by 600 μL of ChIRP biotin elution buffer (12.5 mM biotin, 7.5 mM HEPES, pH 7.9, 75 mM NaCl, 1.5 mM EDTA, 0.15% SDS, 0.075% sarkosyl, and 0.02% Na-Deoxycholate), on rotation at 25°C for 20 min and at 65°C for 15 min shaking. Eluent was transferred and pooled by total twice elution (~1200 μL), and residual beads were removed using the magnetic stand. 25% total volume (300 μL) trichloroacetic acid was added, vortexed, and incubated at 4°C overnight for precipitation. Next day, proteins were pelleted at maximum speed at 4°C for 30 min, washed with 1 mL of cold acetone, and air dried after removing acetone at RT for 1 min. Proteins were solubilized in 10 μL of 1xLDS buffer with 30 mM DTT and boiled at 95°C for 30 min for reverse-crosslinking. Solubilized proteins were prepared for mass spectrometry.

The protein eluate samples were taken the same volume for S-Trap (S-Trap^™^ micro MS sample prep kit, Protifi) procedure as protocol described. Briefly, protein solution was denatured with 10% SDS, reduced with 10 mM final concentration of DTT at 60 °C for 15 minutes and alkylated with 20mM final concentration of IAM at room temperature for 30 minutes. Then, all samples were quenched with 10 mM DTT to eliminate excess IAM in the samples. The protein samples were then acidified with 27.5% phosphoric acid to reach pH ≤ 1. Proteins were trapped into the S-Trap column by centrifuge at 10,000 g for 30 seconds and washed with 100 mM TEAB (final) in 90% methanol repeatedly. 1 μg Trypsin/LysC was added to protein for overnight digestion at 37 °C. Digested samples were quenched with 0.2% formic acid. Samples were then eluted from the S-Trap column with sequential addition and centrifuge of buffer 1 (50 mM TEAB), buffer 2 (0.2% formic acid) and buffer 3 (50% acetonitrile). The eluted solution was pooled and subsequently dried by SpeedVac (SavantTM SpeedVacTM SPD120, Thermo Fisher Scientific).

Each dried sample was resuspended with 80 μL 100mM HEPEs (pH 8.5) with gently vortex and centrifuge. 8 new TMT labels were resuspended with 80 μL ACN, (OptimaTM, LC/MS grade, Fisher ChemicalTM.) and 20 μL of TMT reagent was added to corresponding sample. All samples were incubated in room temperature for 1 hr. 1 μL of each TMT labelled sample was taken out and mixed to perform a label check, which served as a quality control (QC) for efficacy of TMT labeling process. The TMT label check mixture was mixed with LC buffer A (0.1% Formic Acid) and injected into the mass spec. LC/MS run of label check sample indicated the label efficiency is about 99%. TMT labelled samples were mixed and dried down with Thermo SpeedVac. (SpeedVac SPD120, Thermo Fisher Scientific). Combined samples were desalted using C18 stage tips (Cat # PTR-92–05-18, PhyNexus Inc.).

The dried peptides samples were reconstituted with LC Mobile phase A and analyzed by nano flow HPLC (Ultimate 3000, Thermo Fisher Scientific) followed by Orbitrap EclipseTM TribridTM (Thermo Fisher Scientific). Nanospray FlexTM Ion Source (Thermo Fisher Scientific) was equipped with Column Oven (PRSO-V2, Sonation) to heat up the nano column (Aurora Ultimate, 250 mm × 75 μm ID, 1.7 μm C18, IonOpticks) for peptide separation. The nano LC method was water acetonitrile based 120 minutes long with 0.3 μL/min flowrate. All TMT labeled peptides were first engaged on a trap column (Cat. No: 164535, Thermo Fisher Scientific) and then were delivered to the separation nano column by the mobile phase. A TMT specific MS2-based mass spectrometry method on Orbitrap Eclipse was used to sequence TMT peptides that were eluted from the nano column.

The ionized peptides were fractionated by FAIMS Pro^™^ using a 3-CV (−45, −60, −75 V) method. For the full MS, 120,000 resolution was used with the scan range of 400 m/z – 1600 m/z. ‘Standard’ AGC target and ‘Auto’ Maximum Injection Time were selected. For the dd-MS(MS2), resolution was 50,000 and isolation window was 0.7 Da. Normalized AGC Target was set at 250%. Maximum Injection Time Mode was Auto and Collision Energy mode was ‘Fixed’. TMT Quant was performed with Proteome Discoverer 2.5. Raw data was searched using UniProt Homo Sapiens database, Proteome ID UP000005640.

#### RNA-Binding Protein (RBP) Analysis

Enriched RBPs associated with *PVT1-MYC* fusion transcripts were identified from ChIRP-MS data using the following criteria: (1) Signal-to-background filter: Proteins with a fold change (FC) > 1.5 relative to RNase-treated ChIRP negative controls and expression level > 100 were retained to eliminate RNA-independent background interactions. (2) Fusion-specific RBP detection: To identify fusion-specific RBPs in COLO320DM cells, we compared ChIRP-MS ranks for each protein between COLO320DM and COLO320HSR. Proteins were selected if their rank in COLO320DM (*PVT1* exon 1 or *MYC* ChIRP-MS) was at least twofold better (i.e., lower) than their rank in the COLO320HSR *MYC* ChIRP-MS.

Functional annotation and interaction analysis of candidate RBPs were performed using the STRING database (https://string-db.org/).^89^ Protein-protein interactions were sourced from curated databases (BioCarta, BioCyc, GO, KEGG, Reactome) and experimental data. K-means clustering was applied to group RBPs into functionally relevant modules.

To further refine the candidate RBPs involved in *PVT1-MYC* fusion regulation, we intersected three datasets: (1) Enriched RBPs from *PVT1* exon 1 or *MYC* ChIRP-MS in COLO320DM. (2) RBP binding motifs were significantly enriched (p < 0.01) in *PVT1* exon 1, identified using RBPmap (https://rbpmap.technion.ac.il/).^90^ (3) eCLIP peaks from ENCODE with significant enrichment (p < 0.05) at *PVT1* exon 1 in at least one cell line. SRSF1 and SRSF7 were the only RBPs shared across all three datasets. Notably, among the 185 RBPs available in ENCODE eCLIP of HepG2 or K562 cell lines, only SRSF1 consistently showed enrichment at *PVT1* exon 1 in both cell lines.

For visualization of eCLIP read density, the processed eCLIP bigWig tracks of SRSF1 and SRSF7 performed in the K562 and HepG2 cell lines were downloaded from the ENCODE database. eCLIP tracks were visualized using Integrative Genomics Viewer (v2.19.1).

#### AlphaFold3-based structural analysis

A modeling technique which combined *de novo* structure prediction from AlphaFold 3^56^ and manual refinement^100^ was used to develop a three-dimensional structure of the protein-RNA complex of SRSF1 and the exon 1 mRNA of *PVT1*. To begin the structural modeling, the canonical protein sequences of SRSF1 (UniProt: Q07955) was used. Specifically, to constrain the challenge of *de novo* structure prediction and to improve the accuracy, the protein sequence was reduced to only those sub-sequences or domains which were annotated to be interacting with RNA. Thus, for SRSF1 the residues 10–200 was selected as they contained the RNA recognition motifs (RRM), i.e., RRM1 and RRM2 connected by an inter-domain linker. Similarly, the 5’ 80 nt sequence from *PVT1* exon 1 was used as input. The seed used for the prediction was 1234567 and only the default five predicted models were used for further screening. The models were visualized and screened using UCSF ChimeraX,^101^ of which a model that had the best concordance with known annotation was chosen as the representative structure.

#### DeepCLIP score analysis

SRSF1 binding score was calculated by using a deep learning method, DeepCLIP.^58^ This method was trained on eCLIP SRSF1 data from ENCODE-K562 cell line after which the query wildtype and mutant sequences of *PVT1* exon 1, each of length 75 nt were provided. The mutant sequences contained a GGA > UUA substitution.

#### RNA immunoprecipitation (RNA-IP)

For SRSF1 RNA-IP with endogenous RNAs in COLO320DM cells, 10E6 cells were prepared per IP. For SRSF1 RNA-IP with reporter RNAs in HEK293T cells, each 1.2E6 cells were seeded in 10 cm dish 1 day before transfection, transfected with 10 μg of reporter constructs, and collected 2 days post-transfection. 5E6 cells were prepared per IP.

RNA-IP was performed as previously described,^102^ with minor modifications. Cells were harvested at ~70–80% confluency, washed, and lysed in RNA-IP buffer (20 mM HEPES pH 7.5, 100 mM KCl, 0.1 mM EDTA, 0.2% NP-40, 10% glycerol) supplemented with protease/phosphatase inhibitors (ThermoFisher Scientific, 78440) and RNase inhibitor (Invitrogen, AM2694). Lysates were cleared by centrifugation, and 2–4% of lysates were reserved as input for RNA extraction and Western blot (WB). 50 μL of Dynabeads Protein A (Invitrogen, 10001D) were pre-conjugated with 5 μg of either anti-normal rabbit IgG control (Cell Signaling Technology, #2729) or anti-SRSF1 (Bethyl Laboratories, A302–052A) antibodies per IP for 2 hr at 4 °C with rotation. Normal IgG was used as a negative control. After conjugation, beads were washed three times with RNA-IP buffer, and the remaining lysates were incubated with antibody-conjugated beads for 2 hr at 4 °C with rotation. After incubation, beads were washed three times with RNA-IP buffer, and 75% of IP-ed samples were used for RNA extraction and 25% were processed for immunoblotting.

RNA was isolated using TRIzol (Invitrogen, 15596026), followed by the Direct-zol RNA MicroPrep kit (Zymo Research, R2060). For WB, proteins were eluted in LDS buffer (Invitrogen), resolved on 4–12% NuPAGE Bis-Tris gels, transferred to PVDF membranes using the iBlot system (Invitrogen), and immunoblotted by anti-SRSF1 (Thermo Fisher Scientific 32–4500) and anti-alpha TUBULIN antibodies (Abcam, ab7291). And the signals were developed using HRP-conjugated secondary antibodies (Invitrogen, 31430) and Immobilion Western HRP (Milipore, WBLUF0100).

#### MYC rescue assay

Conditional MYC expressing murine hepatocellular carcinoma cells, EC4 cells were seeded at a cell density of 7000 cells per well in 96 well plate. Cells were transfected with the plasmid constructs (200 ng/well) after 24 hr (Day 1). Following transfection, MYC transcription was turned off treating cells with doxycycline (2 ng/mL) after 48 hr of transfection (Day 3). The cell viability was measured using CellTiter-Glo Luminescent Cell Viability Assay (Promega, G7570) to evaluate the rescue effect of the plasmid constructs. The luminescence values were normalized to GFP construct and the relative change in cell viability was calculated. The statistical significance was calculated using ordinary one-way ANOVA in GraphPad Prism 10.2.2.

#### CRISPRi *PVT1* RNA-seq

Lentivirus was generated using the UCOE-SFFV-dCas9-BFP-KRAB construct (Addgene #85969). COLO320DM cells were transduced with the lentivirus, incubated for 2 days, and sorted for BFP-positive cells via flow cytometry. Polyclonal BFP-positive cells were subsequently monocloned to isolate clones with stable and homogenous expression of dCas9-BFP-KRAB and high CRISPR interference (CRISPRi) efficiency. The guide RNAs for *PVT1* (sgPVT1) and non-targeting control (sgNTC) were used as described in Hung et al.^4^

COLO320DM dCas9-KRAB BFP cells were plated 24 hr prior to transduction. The cells were transduced with either sgPVT1 or sgNTC. On Day 1, 24 hr post-transduction, puromycin was added to the culture medium at a concentration of 2 μg/mL to initiate the selection process. On Day 3, 72 hr post-transduction, the cells were collected, washed in PBS, and analyzed using an Attune flow cytometer to quantify the expression and cellular effects of the sgRNA targeting. On Day 4, 96 hr post-transduction, the cells were harvested, and RNA was extracted for bulk RNA sequencing. RNA libraries were constructed using TruSeq Stranded Total RNA Library Prep Kit with Ribo-Zero (Illumina, catalog no. 20020596) and sequenced by Nextseq 550 (Illumina), 75 bp paired-end reads per sample.

#### CRISPRi *PVT1* RNA-seq analysis

Raw paired-end RNA-seq fastq files were aligned to the GRCh38 genome reference using STAR (version 2.7.10b). Following alignment, read counts for each gene were obtained using featureCounts (version 2.0.8) for subsequent analyses. From these read counts, differentially expressed genes between CRISPRi sgPVT1 and sgNTC (non-targeting control) COLO320DM cells were identified using the DESeq2 R package (version 1.44.0). The gene list, ordered by log2 fold change as calculated by DESeq2, was then used for Gene Set Enrichment Analysis (GSEA) with the GSEA function from the clusterProfiler R package (version 4.12.2), employing human hallmark gene sets from the msigdbr R package. Significantly enriched pathways were defined as those with adjusted p-values less than 0.05, adjusted using the Benjamini-Hochberg method.

Expression levels of canonical *MYC* and *PVT1-MYC* were assessed by FPKM (Fragments Per Kilobase of transcript per Million read). Reads that were aligned to *MYC* exon 1 or those spanning *MYC* exon 1–2 were classified to canonical *MYC* transcripts while reads spanning *PVT1* and *MYC* exon 2–3 were classified to *PVT1-MYC*. Assigned read counts were normalized by the length of canonical *MYC* and *PVT1-MYC* transcripts, respectively and total read counts per million. Following this, we normalized RNA expression levels of canonical *MYC* and *PVT1-MYC* FPKM in CRISPRi sgPVT1 divided by corresponding sgNTC to evaluate relative expression changes.

### QUANTIFICATION AND STATISTICAL ANALYSIS

All statistical analyses and quantification were conducted by R or GraphPad Prism 10 software. Differences between groups were evaluated by two-tailed t-test, one-way ANOVA or Wilcoxon rank-sum-test. All n numbers indicated in the figure legends unless otherwise stated, represent biological replicates. Bar graph data are represented as mean ± SEM from at least three biological replicates. Details of exact statistical analysis, tests, and other information can be found in the figure legends and Methods. Figure legends provide the statistical details of the experiments and assays, including the number of replicates (n), statistical tests, comparisons and *p*-values used in each figure.

## Supplementary Material

Table S3

Table S4

Table S2

Table S5

Table S1

6

SUPPLEMENTAL INFORMATION

Supplemental information can be found online at https://doi.org/10.1016/j.cell.2025.12.009.

## Figures and Tables

**KEY RESOURCES TABLE:** 

REAGENT or RESOURCE	SOURCE	IDENTIFIER
Antibodies		
Rabbit polyclonal anti-SRSF1	Bethyl Laboratories	RRID: AB_1604258
Mouse monoclonal anti-alpha Tubulin [DM1A]	Abcam	RRID: AB_2241126
Bacterial and virus strains		
COLO320DM dCas9-BFP-KRAB (CRISPRi) cell line	This paper	N/A
COLO320DM CRISPRi PVT1 cell line	This paper	N/A
Chemicals, peptides, and recombinant proteins		
Liberase	Millipore Sigma	5401020001
KaryoMAX^™^ Colcemid^™^	Thermo Fisher Scientific	15212012
Actinomycin D	Sigma-Aldrich	SBR00013
Cycloheximide	Sigma-Aldrich	C4859
SMG1 inhibitor	Sigma-Aldrich	SML3864
Lipofectamine 3000 Transfection Reagent	Thermo Scientific	L3000008
RNase A	Fisher Scientific	12-091-021
Doxycycline	Sigma-Aldrich	D9891
Critical commercial assays		
10x Genomics Chromium Fixed RNA Profiling kit	10x Genomics	Human Transcriptome 4 rxns x 4 barcode 1000414
Direct-zol^™^ RNA Purification miniprep Kit	Zymo Research	R2052
Direct-zol^™^ RNA Purification microprep Kit	Zymo Research	R2060
Qubit dsDNA Quantitation, High-Sensitivity kit	Thermo Fisher	Q32851
High-Sensitivity D1000 TapeStation	Agilent	5067-5585
Direct RNA Sequencing Ligation Kit	Oxford Nanopore	SQK-RNA002
MagAttract HMW DNA kit	Qiagen	67563
Ligation Sequencing DNA kit V14	Oxford Nanopore	SQK-LSK114
TruSeq Stranded Total RNA Library Prep Kit with Ribo-Zero	Illumina	20020596
RNeasy Plus mini Kit	Qiagen	74136
Primescript^™^ RT Reagent Kit with gDNA Eraser	Takara Bio	RR047A
Maxwell RSC simplyRNA Cells Kit	Promega	AS1390
Brilliant II SYBR Green QRT-PCR 1-Step Master Mix	Agilent Technologies	600825
CellTiter-Glo Luminescent Cell Viability Assay	Promega	G7570
Deposited data		
Raw Flex scRNA-seq	This paper	NCBI BioProject: PRJNA1346268
CRISPRi *PVT1* RNA-seq	This paper	NCBI BioProject: PRJNA1346268
Long-read RNA sequencing	This paper	NCBI BioProject: PRJNA1346268
Long-read DNA sequencing	This paper	NCBI BioProject: PRJNA1346268
CCLE AmpliconArchitect outputs	Amplicon Repository	https://ampliconrepository.org/project/6580f373ea940f33361428ba
TCGA AmpliconArchitect outputs	Amplicon Repository	https://ampliconrepository.org/project/655bddb5bba7c92509525039
CCLE Structural variation	CCLE_translocations_SvABA_20181221	https://depmap.org/portal/data_page/?tab=allData&releasename=CCLE%202019&filename=CCLE_translocations_SvABA_20181221.xlsx
TCGA Structural variation	https://portal.gdc.cancer.gov/	TCGA SvABA somatic structural variation
CCLE RNA fusion	DepMap Public 24Q2	https://depmap.org/portal/data_page/?tab=allData&releasename=DepMap%20Public%2024Q2&filename=OmicsFusionUnfilteredProfile.csv
TCGA RNA fusion	Haas et al.^25^	https://www.sciencedirect.com/science/article/pii/S2667237523000863?via%3Dihub#mmc3
Known fusion library	CTAT_HumanFusionLib	https://data.broadinstitute.org/Trinity/CTAT_RESOURCE_LIB/GRCh38_gencode_v37_CTAT_lib_Mar012021.plug-n-play.tar.gz
NCBI Human Genome Annotation Features (GTF)	GRCh38.p14	https://www.ncbi.nlm.nih.gov/datasets/genome/GCF_000001405.40/
TCGA RNA-seq	UCSC TOIL RSEM TPM	https://xenabrowser.net/datapages/?dataset=tcga_RSEM_gene_tpm&host=https%3A%2F%2Ftoil.xenahubs.net&removeHub=http%3A%2F%2F127.0.0.1%3A7222
CCLE RNA-seq	CCLE_RNAseq_rsem_genes_tpm_20180929.txt	https://depmap.org/portal/data_page/?tab=allData&releasename=CCLE%202019&filename=CCLE_RNAseq_rsem_genes_tpm_20180929.txt.gz
eCLIP	ENCODE RNA-protein interactions (ENCORE)	https://www.encodeproject.org/search/?type=Experiment&status=released&internal_tags=ENCORE&assay_title=eCLIP&files.file_type=bed+narrowPeak
Original western blot images	This paper; Mendeley Data	https://data.mendeley.com/datasets/cnz75cv3cs/2
Source imaging data	This paper; Stanford Digital Repository	https://doi.org/10.25740/cp783mb3217
Experimental models: Cell lines		
COLO320DM dCas9-BFP-KRAB (CRISPRi) cell line	This paper	N/A
Experimental models: Organisms/strains		
COLO320DM/HSR xenograft mice (Female Athymic Nude Foxn1nu Mice)	This paper	Charles Rivers Laboratories
Oligonucleotides		
See Table S3 for oligo sequences	This paper	N/A
Recombinant DNA		
pGL4-PP-PE1-ME2-3-Flag-mNG11_TK-mCherry	This paper	N/A
pGL4-PP- ME1-ME2-3-Flag-mNG11_TK-mCherry	This paper	N/A
pGL4-EF1α-PE1-ME2-3-Flag-mNG11_TK-mCherry	This paper	N/A
pGL4-EF1α-ME1-ME2-3-Flag-mNG11_TK-mCherry	This paper	N/A
pGL4- EF1α-PE1-ME2-3 Del1-Flag-mNG11_TK-mCherry	This paper	N/A
pGL4- EF1α-PE1-ME2-3 Del2-Flag-mNG11_TK-mCherry	This paper	N/A
pGL4- EF1α-PE1-ME2-3 Del3-Flag-mNG11_TK-mCherry	This paper	N/A
pGL4- EF1α-PE1-ME2-3 Del4-Flag-mNG11_TK-mCherry	This paper	N/A
pGL4- EF1α-PE1-ME2-3 pMut-Flag-mNG11_TK-mCherry	This paper	N/A
pGL4-PP-PE1-Nluc_TK-Fluc	Hung et al.^4^	N/A
pGL4-PP-Nluc_TK-Fluc	This paper	N/A
pGL4-minP-PE1-Nluc_TK-Fluc	This paper	N/A
pGL4-minP-Nluc_TK-Fluc	This paper	N/A
Software and algorithms		
Cellranger v8.0.0	10x Genomics	https://www.10xgenomics.com/support/software/cell-ranger/8.0
Seurat v4.3.0	Hao et al.^82^	https://satijalab.org/seurat/
STAR 2.7.10b	Dobin et al.^83^	https://github.com/alexdobin/STAR
featureCounts 2.0.8	Liao et al.^84^	https://subread.sourceforge.net/featureCounts.html
Jaffa 2.3	Davidson et al.^85^	https://github.com/Oshlack/JAFFA
Guppy 2.3.7	Oxford Nanopore Technologies	https://nanoporetech.com/software/other/guppy/
Dorado 0.2.4	Oxford Nanopore Technologies	https://github.com/nanoporetech/dorado
Sniffles 1.0.11	Smolka et al.^86^	https://github.com/fritzsedlazeck/Sniffles
NGMLR 0.2.7	Sedlazeck et al.^87^	https://github.com/philres/ngmlr
CoRAL	Zhu et al.^72^	https://github.com/AmpliconSuite/CoRAL
R 4.4.1	R Core Team (2024)	https://www.r-project.org/
Python 3.12.2	Van Rossum and Drake (2009)	https://www.python.org
Prism 10	GraphPad	https://www.graphpad.com/scientific-software/prism/
Metascape	Zhou et al.^88^	https://metascape.org/gp/index.html#/main/step1
STRING Version: 12.0	Szklarczyk et al.^89^	https://string-db.org/
RBPmap	Paz et al.^90^	https://rbpmap.technion.ac.il/index.html
Biorender	Biorender	https://www.biorender.com

## Highlights

- EcDNA serves as a major platform for oncogene fusion formation across cancers
- EcDNA-borne oncogene fusions display tissue specificity and diagnostic potential
- The prevalent *PVT1* exon 1 fusion on ecDNA stabilizes partner RNAs via SRSF1
- *PVT1-MYC* fusion enhances MYC transcriptional output and cancer cell survival
