# Supplementary material for: EcDNA-borne structural variants drive oncogenic fusion transcript amplification
Source: Cell. Author manuscript; Available in PMC 2026 Jul 26. (PMC13402033; doi:10.1016/j.cell.2025.12.009)
Supplement: 6 [file NIHMS2185007-supplement-6.pdf]

Supplemental figures

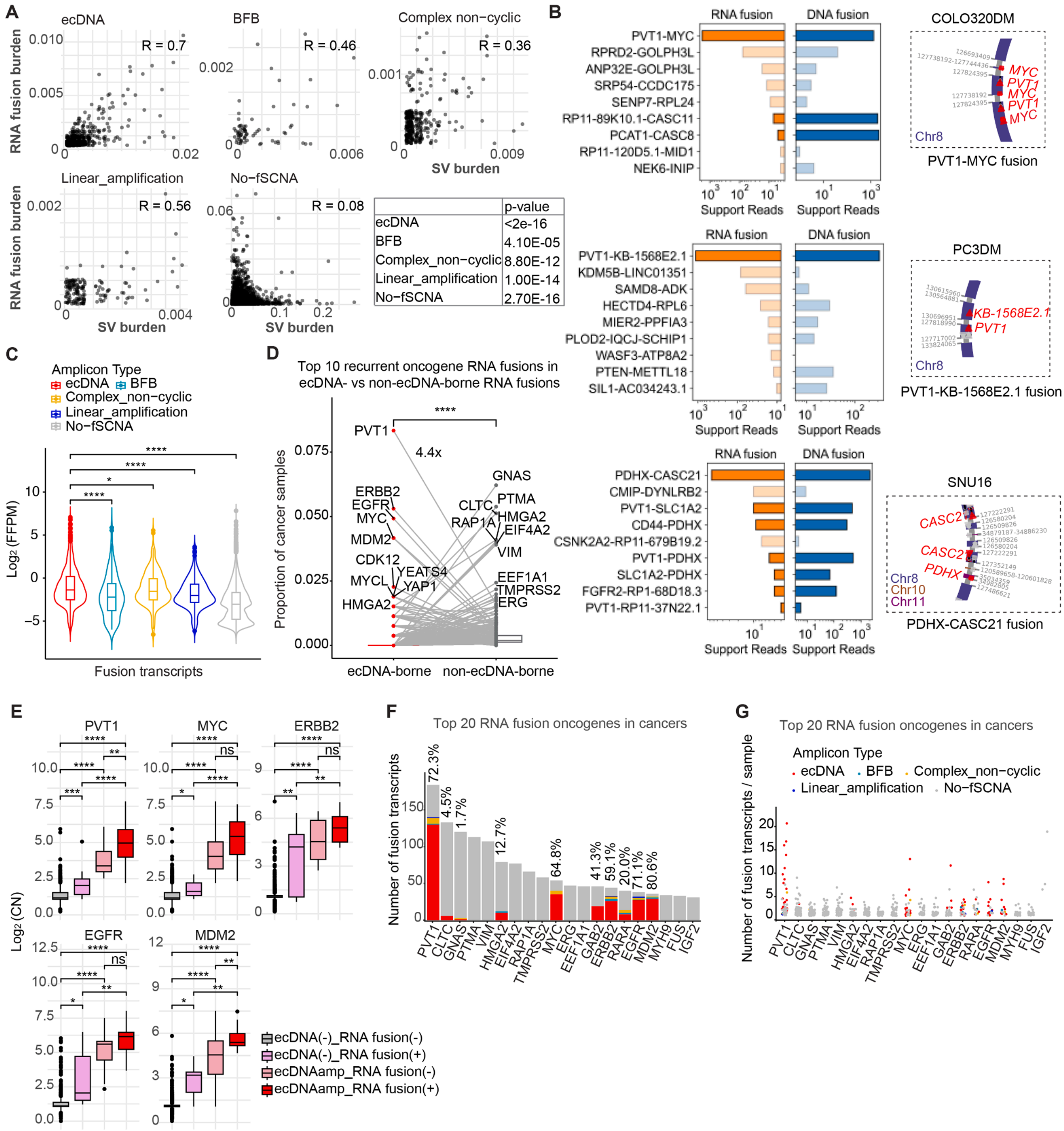

Figure S1. Landscape of gene fusions on ecDNAs, related to Figure 1

(A) Correlation between SV and RNA fusion burden by amplicon type. Each dot represents a 100 kb genomic window, categorized by amplicon type. Correlation efficiency was determined by Pearson's correlation coefficient (*R*).

(B) RNA and DNA fusion detection by long-read sequencing. Fusion genes expressing the top 9 most abundant fusion transcripts in each ecDNA(+) cell line are presented. Left: the number of RNA fusion and DNA fusion reads. EcDNA-amplified genes are denoted as dark-colored and non-ecDNA genes as light-colored bars. Right: the genomic structures of ecDNA supporting the most abundant RNA fusions.

(legend continued on next page)

(C) Expression level (log2FFPM) of fusion transcripts by amplicon type.
(D) Proportion of cancer samples with oncogene RNA fusions. Red dots indicate genes with ecDNA-borne RNA fusions; gray dots, genes with non-ecDNA-borne RNA fusions. The top 10 recurrent oncogenes are labeled for each group, and shared oncogenes are connected by lines.
(E) DNA copy-number distribution of representative oncogenes with ecDNA-borne RNA fusions, grouped by RNA fusions and ecDNA amplification status.
(F) Number of fusion transcript species for the top 20 RNA fusion oncogenes across cancer samples. Amplicon types are color-coded in bars, with the percentage of ecDNA-borne RNA fusions indicated at the top of each bar.
(G) Number of different fusion transcript species per cancer sample for the top 20 RNA fusion oncogenes shown in (F). Each dot represents a cancer sample harboring the corresponding oncogene RNA fusion, with amplicon type color-coded.
(A and C–G) Samples are from TCGA and CCLE.
(C, D, and E) $p > 0.05$ (ns), *$p < 0.05$, **$p < 0.01$, ***$p < 0.001$, ****$p < 0.0001$ by two-tailed unpaired t test.

CellPress
OPEN ACCESS

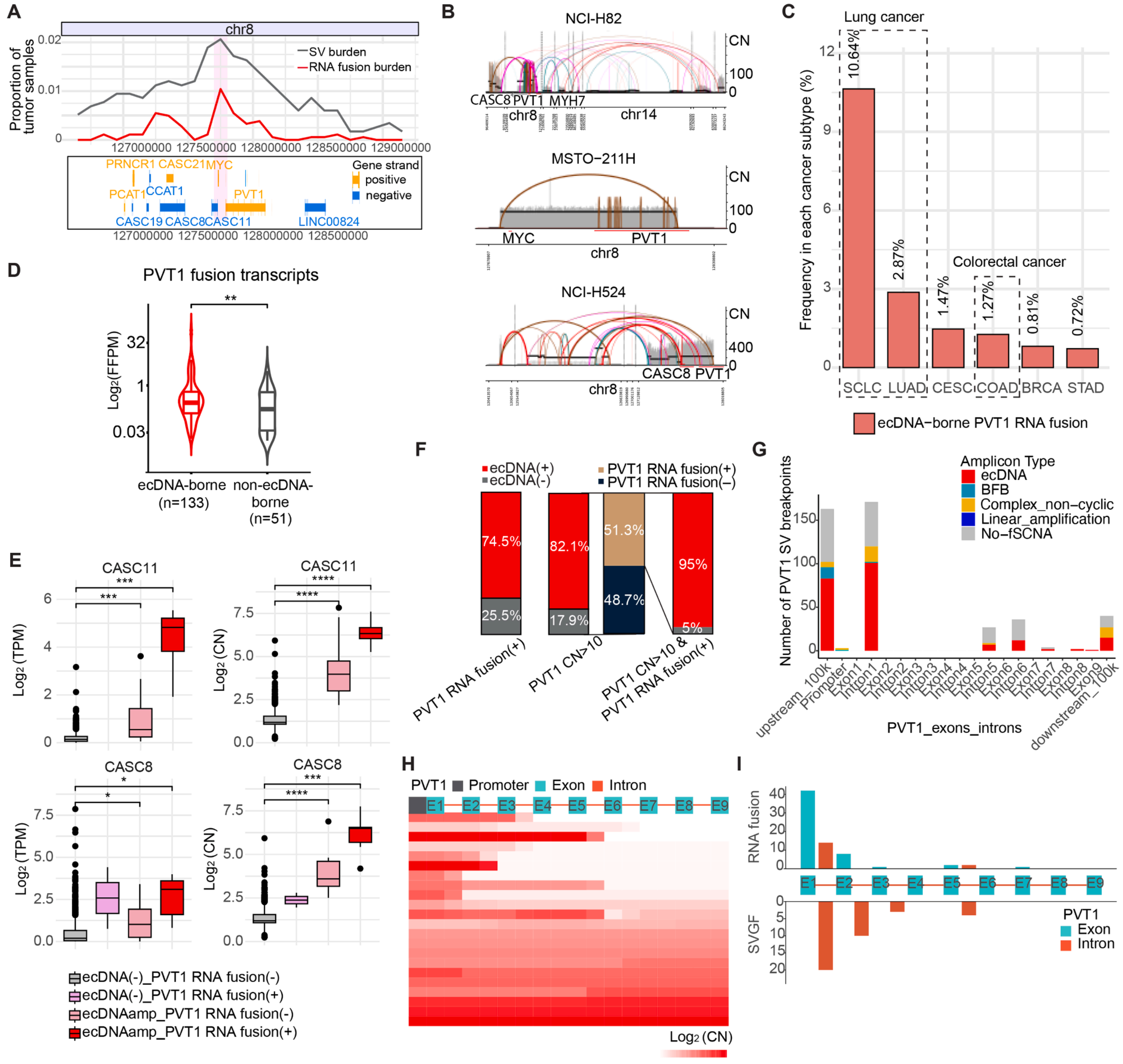

Figure S2. Landscape of ecDNA-borne *PVT1* fusions, related to Figure 2

(A) Distribution of SV and RNA fusion burden peaks near the *MYC/PVT1* region in ecDNA(+) cancers.

(B) Genomic rearrangements on the *PVT1* locus generating multiple gene fusions in ecDNA(+) lung cancer cell lines by AmpliconArchitect analysis. Gray bars indicate copy number, and discordant genomic segments with SVs relate to colorful arches.

(C) Percentage of samples with ecDNA-borne *PVT1* RNA fusions in each cancer subtype. SCLC, small cell lung cancer; LUAD, lung adenocarcinoma; CESC, cervical squamous cell carcinoma and endocervical adenocarcinoma; COAD, colon adenocarcinoma; BRCA, breast invasive carcinoma; STAD, stomach adenocarcinoma.

(D) Expression level (log2 FFPM) of ecDNA-borne and non-ecDNA-borne *PVT1* RNA fusions. The numbers of *PVT1* fusion transcripts used in analysis are indicated in parentheses.

(E) RNA expression levels of *CASC11* and *CASC8* genes, grouped by *PVT1* RNA fusions and ecDNA amplification status.

(F) Proportion of ecDNA (+) cancer samples compared among three groups: cancers with *PVT1* fusion transcripts (*PVT1* RNA fusion [+]), cancers with *PVT1* copy-number variation (copy number [CN] > 10), and cancers with both *PVT1* CN > 10 and *PVT1* RNA fusion.

(G) Structural variant (SV) breakpoint distribution within the *PVT1* locus. The *x* axis spans 100 kb upstream and downstream of the *PVT1* gene body, including the promoter (1 kb upstream of transcription start site [TSS]), exons, and introns. The *y* axis indicates the number of SV breakpoints. Colors denote amplicon types.

(H) Copy-number distribution of the *PVT1* locus in samples with ecDNA-borne *PVT1* RNA fusions. Heatmap columns represent the *PVT1* promoter (1 kb upstream of TSS), exons, and introns. Rows correspond to cancer samples. Colors denote log2(copy number), with darker red indicating higher copy number.

(legend continued on next page)

(I) Distribution of RNA fusion breakpoints (top) and SVGF breakpoints (bottom) of *PVT1* fusions across exonic and intronic regions of *PVT1*, measured by long-read RNA and DNA sequencing in ecDNA(+) cell line models and their isogenic pairs where *PVT1* fusion transcripts were detected (COLO320DM, COLO320HSR, PC3DM, SNU16, GBM39KT, and GBM39HSR).
(A–H) Samples are from TCGA and CCLE.
(D and E) $^{*}p < 0.05$, $^{**}p < 0.01$, $^{***}p < 0.001$, $^{****}p < 0.0001$ by two-tailed unpaired t test.

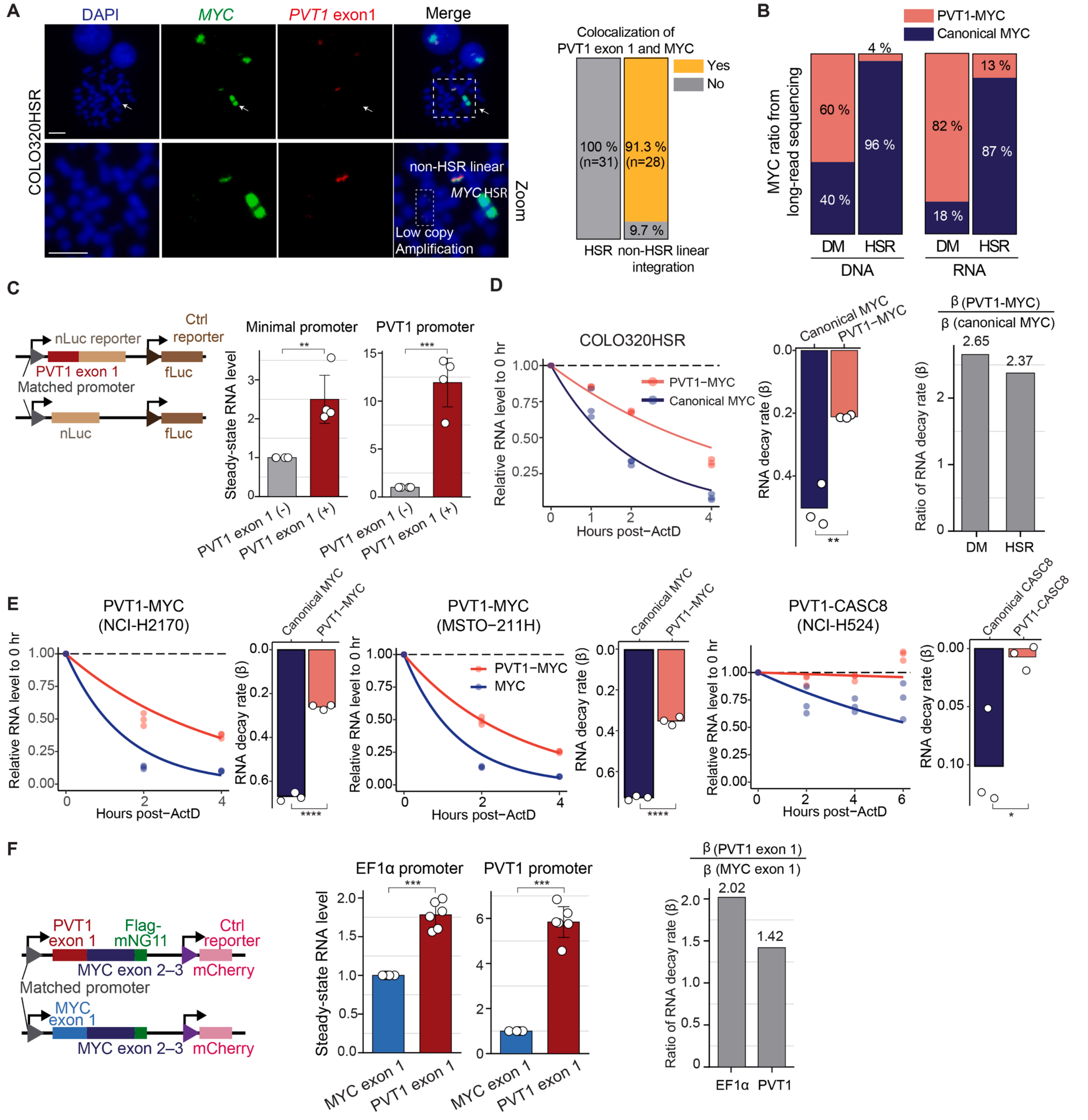

Figure S3. *PVT1* exon 1 fusion enhances RNA stability, related to Figure 3

(A) Left: magnified DNA FISH images of the COLO320HSR cell line in Figure 3B. Right: quantification of colocalized signals between *PVT1* exon 1 and *MYC* on HSR and non-HSR linear integration from DNA FISH images of the COLO320HSR cell line (n = 31).

(B) Proportion of DNA copy number (left) and RNA abundance (right) of *PVT1-MYC* and canonical *MYC* in COLO320DM and COLO320HSR cell lines, measured by long-read DNA and RNA-seq, respectively.

(C) Steady-state reporter RNA levels in COLO320DM cells (n = 4). RNA levels were measured by RT-qPCR and normalized to *PVT1* exon 1(−) reporter and fLuc internal control RNA. Left: schematic of the reporter constructs with or without *PVT1* exon 1 fusion. Right: steady-state RNA levels of reporter transcripts. Data are represented as mean ± SEM.

(D) RNA stability and decay rates of endogenous *PVT1-MYC* and canonical *MYC* transcripts in COLO320HSR cells (n = 3). Right: fold change in RNA decay rates between *PVT1-MYC* and canonical *MYC* transcripts in COLO320DM (from Figure 3F) and COLO320HSR cells.

(E) RNA stability and decay rates of endogenous *PVT1*-fusion and canonical transcripts in multiple cell lines (n = 3).

(legend continued on next page)

(F) Steady-state reporter RNA levels and fold change in RNA decay rates of reporter transcripts in COLO320DM cells (n = 6). Left: schematic of the reporter constructs. Middle: steady-state reporter RNA levels at ActD 0 h measured by RT-qPCR and normalized to the *MYC* exon 1-fused reporter and mCherry internal control RNA. Data are represented as mean ± SEM. Right: fold change in RNA decay rates between the *PVT1* exon 1- and *MYC* exon 1-fused reporters.

(C–F) *$p < 0.03$, **$p < 0.002$, ***$p < 0.0002$, ****$p < 0.0001$ by two-tailed t test.

(D and E) RNA abundance was measured by RT-qPCR and normalized to 0 h and *GAPDH* internal control RNA.

CellPress
OPEN ACCESS

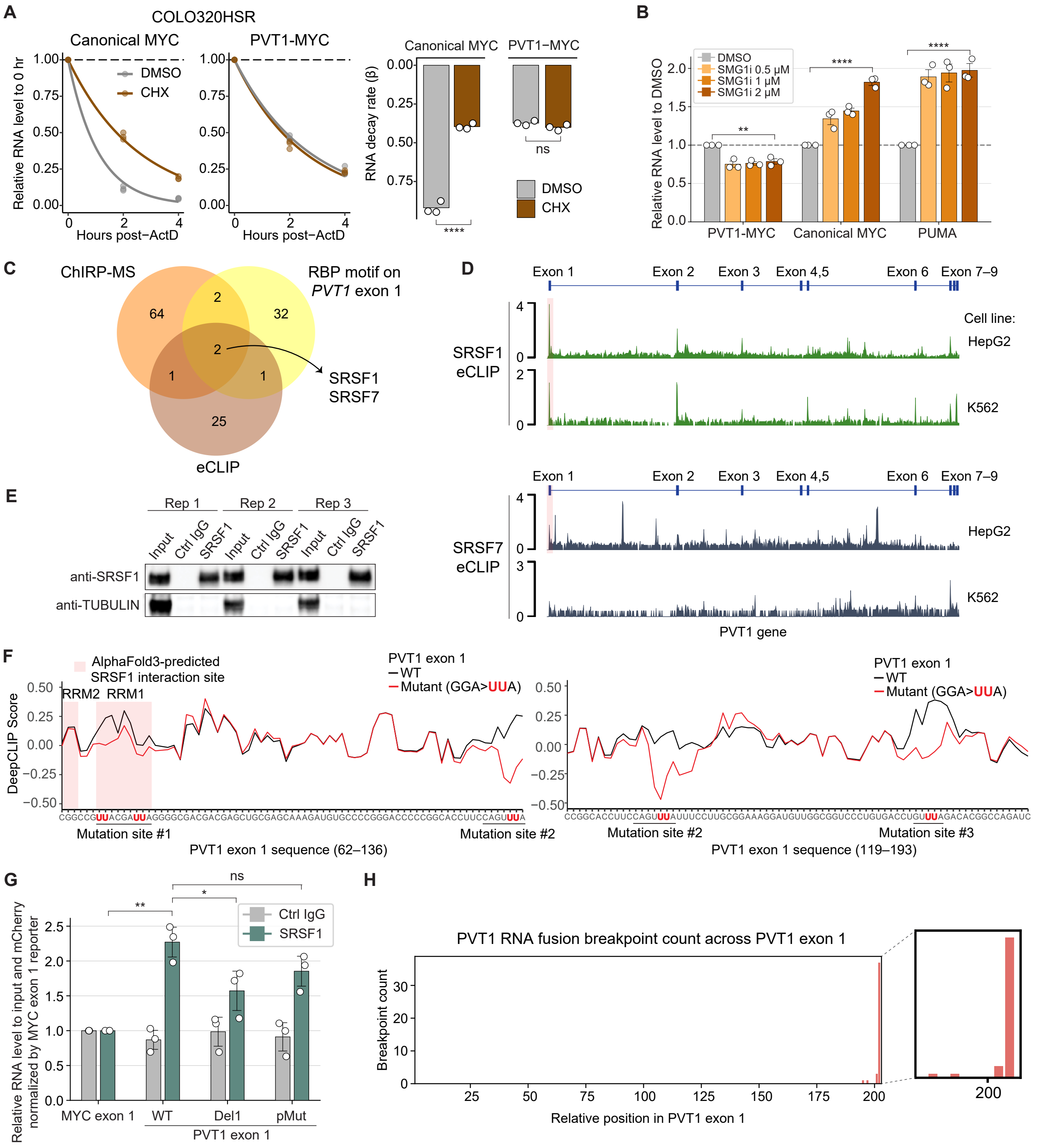

Figure S4. SRSF1 binding contributes to *PVT1* exon 1-mediated RNA stabilization, related to Figure 4

(A) RNA stability and decay rates of endogenous *PVT1-MYC* and canonical *MYC* mRNAs upon translation inhibition with cycloheximide (CHX) in COLO320HSR cells as described in Figure 4D ($n = 3$).

(B) Steady-state RNA levels of endogenous *PVT1-MYC* and canonical *MYC* transcripts upon NMD inhibition with SMG1i in COLO320DM cells ($n = 3$). Each dot represents relative RNA abundance normalized to DMSO and *GAPDH* internal control RNA. *PUMA* mRNA, a known NMD target, was used as a positive control. Data are represented as mean ± SEM.

(C) Venn diagram showing intersections among three datasets (*PVT1-MYC* ChIRP-MS hits in COLO320DM, eCLIP, and RBPmotif in *PVT1* exon 1), highlighting SRSF1 and SRSF7 as shared candidate RBPs.

(legend continued on next page)

(D) Read density tracks of SRSF1 and SRSF7 eCLIP across exonic and intronic regions of *PVT1* in HepG2 and K562 cell lines, with an enriched peak at *PVT1* exon 1 highlighted in a pink box.
(E) Western blot of SRSF1 RNA-IP in COLO320DM ($n = 3$).
(F) DeepCLIP score distribution along the *PVT1* exon 1 mRNA sequence comparing the wild-type and mutant sequences (pMut). GGA > UUA substitutions in the mutation sites (underlined) are denoted in red.
(G) Reporter RNA enrichment of SRSF1 RNA-IP in HEK293T cells ($n = 3$). RNA enrichment was calculated relative to input reporter RNA levels and mCherry internal negative control. Fold changes relative to the *MYC* exon 1-fused reporter are presented. Data are represented as mean ± SEM.
(H) Distribution of RNA fusion breakpoints across *PVT1* exon 1, measured by long-read RNA-seq in ecDNA(+) cell line models and their isogenic pairs where *PVT1* fusion transcripts are detected (COLO320DM, COLO320HSR, PC3DM, SNU16, GBM39KT, and GBM39HSR).
(A, B, and G) RNA levels were measured by RT-qPCR, $p > 0.03$ (ns), $^{*}p < 0.03$, $^{**}p < 0.002$, $^{***}p < 0.0002$, $^{****}p < 0.0001$ by two-tailed t test.

CellPress
OPEN ACCESS

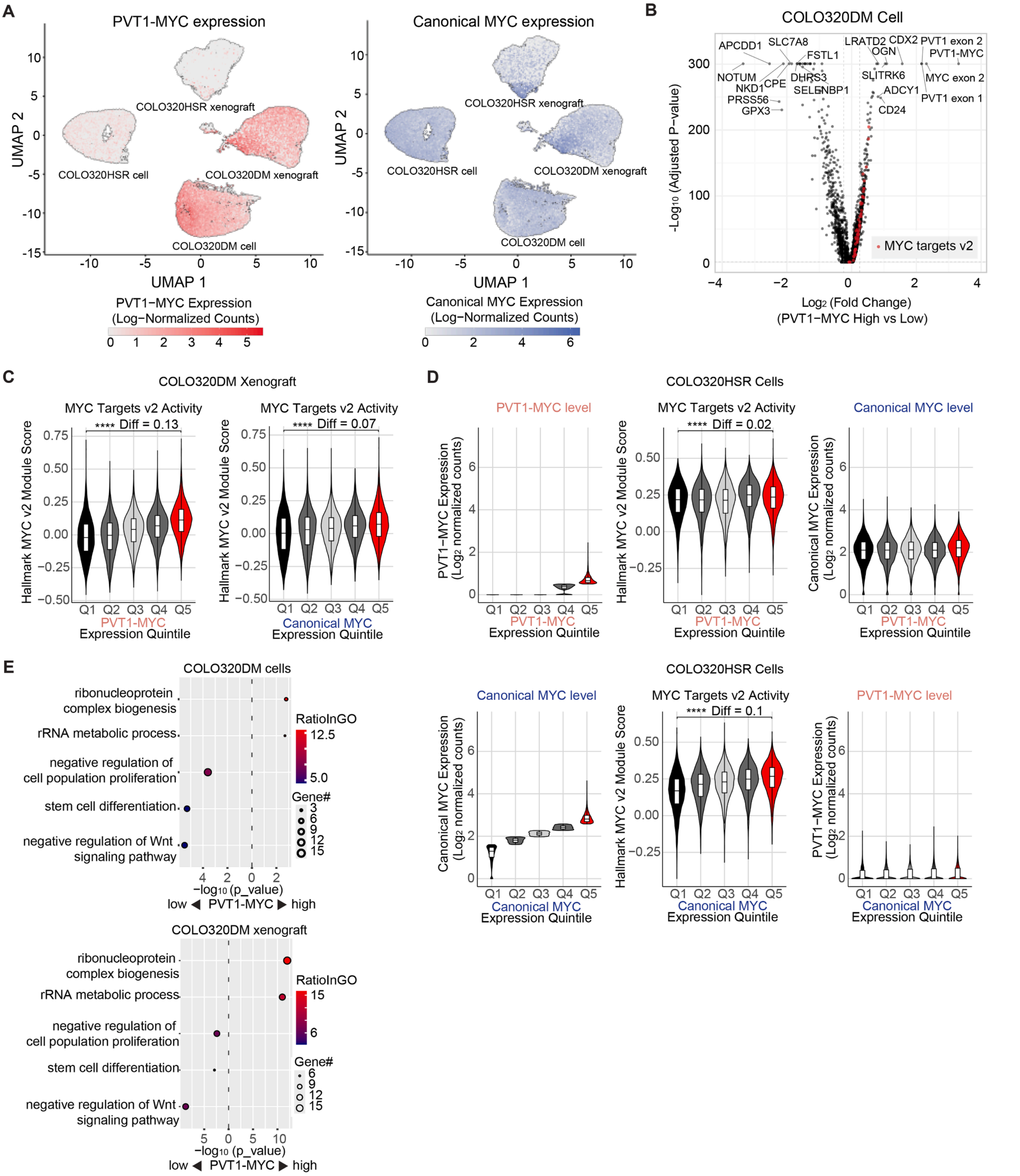

Figure S5. ***PVT1*** **fusion enhances the oncogenic function of** ***MYC*****, related to Figure 5**

(A) UMAP (uniform manifold approximation and projection) visualization of single cells analyzed by Flex scRNA-seq in COLO320DM and COLO320HSR cells and xenograft mice *in vivo* models. Single-cell expression levels of *PVT1-MYC* (left) and canonical *MYC* (right) are shown as red and blue, respectively. Each dot represents a single cell.

(legend continued on next page)

(B) Differentially expressed genes (DEGs) in *PVT1-MYC* high versus low COLO320DM cells, with the top 10 upregulated and downregulated genes labeled. MYC targets v2 genes are highlighted as red dots.

(C) Violin plots displaying dose-dependent MYC targets v2 activity across expression quintiles of *PVT1-MYC* (left) and canonical *MYC* (right) in COLO320DM xenograft. The difference (Diff) was calculated as the mean Hallmark MYC v2 module score of the highest quintile (Q5) minus that of the lowest quintile (Q1) (****p < 0.0001; Wilcoxon rank-sum test).

(D) Violin plots displaying dose-dependent MYC targets v2 activity and expression level of *MYC* isoforms across expression quintiles of *PVT1-MYC* (top) and canonical *MYC* (bottom) in COLO320HSR cells. Diff was calculated as described in (C) (****p < 0.0001; Wilcoxon rank-sum test).

(E) Gene Ontology (GO) analysis of *PVT1-MYC* high (Q5) versus low cells (Q1) in Flex scRNA-seq. GO terms are shown as dot plots, where dot size represents the number of genes and color indicates the proportion of DEGs within each term. Top: COLO320DM cells; bottom: COLO320DM xenografts.
